# Bill size variation in northern cardinals associated with anthropogenic drivers across North America

**DOI:** 10.1002/ece3.4038

**Published:** 2018-04-17

**Authors:** Colleen R. Miller, Christopher E. Latimer, Benjamin Zuckerberg

**Affiliations:** ^1^ Department of Forest and Wildlife Ecology University of Wisconsin‐Madison Madison WI USA

**Keywords:** Allen's rule, bill size, climate change, housing density, microevolution, morphology

## Abstract

Allen's rule predicts that homeotherms inhabiting cooler climates will have smaller appendages, while those inhabiting warmer climates will have larger appendages relative to body size. Birds’ bills tend to be larger at lower latitudes, but few studies have tested whether modern climate change and urbanization affect bill size. Our study explored whether bill size in a wide‐ranging bird would be larger in warmer, drier regions and increase with rising temperatures. Furthermore, we predicted that bill size would be larger in densely populated areas, due to urban heat island effects and the higher concentration of supplementary foods. Using measurements from 605 museum specimens, we explored the effects of climate and housing density on northern cardinal bill size over an 85‐year period across the Linnaean subspecies’ range. We quantified the geographic relationships between bill surface area, housing density, and minimum temperature using linear mixed effect models and geographically weighted regression. We then tested whether bill surface area changed due to housing density and temperature in three subregions (Chicago, IL., Washington, D.C., and Ithaca, NY). Across North America, cardinals occupying drier regions had larger bills, a pattern strongest in males. This relationship was mediated by temperature such that birds in warm, dry areas had larger bills than those in cool, dry areas. Over time, female cardinals’ bill size increased with warming temperatures in Washington, D.C., and Ithaca. Bill size was smaller in developed areas of Chicago, but larger in Washington, D.C., while there was no pattern in Ithaca, NY. We found that climate and urbanization were strongly associated with bill size for a wide‐ranging bird. These biogeographic relationships were characterized by sex‐specific differences, varying relationships with housing density, and geographic variability. It is likely that anthropogenic pressures will continue to influence species, potentially promoting microevolutionary changes over space and time.

## 1 INTRODUCTION

For over 150 years, biogeographers have codified their observations of the natural world in a set of rules explaining variability in species’ traits. Biogeographic principles such as Gloger's rule (Gloger, 1833), Allen's rule (Allen, 1877), Bergmann's rule (Blackburn, Gaston, & Loder, 1999; Gardner, Peters, Kearney, Joseph, & Heinsohn, 2011; Scholander, 1954), and Rapoport's rule (Rapoport, 1982) documented broad latitudinal differences in animal pigmentation, appendage size, body size, and range characteristics. These principles describe not only the geographic variation in traits among species, but also variation across species’ populations, and serve as a lasting framework for exploring trait diversity across the world.

Given the latitudinal nature of many biogeographic principles, climate is considered the most likely mechanism underlying variability in species’ traits. For example, Allen's rule predicts that homeotherms with relatively smaller appendages (high volume‐to‐surface ratio) can minimize heat loss and persist in colder climates, while species with larger appendages (low volume‐to‐surface ratio) have more efficient heat dissipation in warmer environments (Allen, 1877). Although the mechanism of thermal adaptation as a driver of appendage size variation has received mixed empirical support (Alho et al., 2011; Nudds & Oswald, 2007), Allen's rule is often invoked as a possible explanation for geographic variation in bill size for birds (Danner, Greenberg, & Sillett, 2014; Friedman, Harmáčková, Economo, & Remeš, 2017; Greenberg & Danner, 2012). Avian bills play an important function in thermoregulation due to vascularized tissue mediating heat transfer and mitigating water loss (Danner et al., 2017; Tattersall, Andrade, & Abe, 2009; Tattersall, Arnaout, & Symonds, 2016), and bill size tends to correlate with climatic gradients both within and across species (Symonds & Tattersall, 2010). For example, populations of saltmarsh sparrows (*Ammodramus caudacutus*) (Greenberg, Danner, Olsen, & Luther, 2012) and song sparrows (*Melospiza melodia*) (Danner & Greenberg, 2015) inhabiting warmer regions have larger bills compared to populations in colder regions.

The relationship between bill size and climate is complicated, and differences in aridity and minimum or maximum temperature can modify the role of bills in thermal adaptation. Patterns in the relationship between bill size and temperature often interact with aridity: The positive relationship between bill size and temperature is amplified in more arid environments (Campbell‐Tennant, Gardner, Kearney, & Symonds, 2015; Greenberg & Danner, 2012). This could be because birds lack sweat glands and the passive dissipation of dry heat by bills could substantially reduce the amount of respiratory water lost by active means such as gular flutter and panting (Greenberg, Cadena, Danner, & Tattersall, 2012; Van de Ven, Martin, Vink, McKechnie, & Cunningham, 2016). In more temperate environments, minimum and maximum temperatures can influence morphology through their effects on thermal metabolism (Campbell‐Tennant et al., 2015; Fristoe et al., 2015; Root, 1988b), and bills can function as either heat dissipaters in hot climates (Symonds & Tattersall, 2010; Tattersall et al., 2009; Van de Ven et al., 2016) or heat conservers in colder climates (Danner & Greenberg, 2015; Hagan & Heath, 1980).

In recent years, there has been a renewed interest in Allen's rule due to the microevolutionary implications of modern climate change. Modern climate change has resulted in unprecedented shifts in temperature and precipitation throughout the globe (IPCC Report 2014). In North America, climate change has and will continue to impact climate patterns, as global temperatures have increased approximately 1.5°C over the past 100 years (Melillo, Richmond, & Yohe, 2014). As a result, whether changes in global climate could promote changes in species’ traits remains a question (Gienapp, Teplitsky, Alho, Mills, & Merilä, 2008). While many studies have focused on Allen's rule over space (Danner & Greenberg, 2015; Friedman et al., 2017; Greenberg & Danner, 2012; Greenberg, Danner et al., 2012; Greenberg, Cadena et al., 2012; Symonds & Tattersall, 2010), few have evaluated changes in bill size over time (Campbell‐Tennant et al., 2015). In one study on Australian parrots, the authors found evidence that bill sizes increased over time, putatively as an adaptation to thermal stress of rising maximum temperatures (Campbell‐Tennant et al., 2015). Many other studies have focused on climate‐induced changes over time in traits such as plumage coloration (Evans & Gustafsson, 2017; Karell, Ahola, Karstinen, Valkama, & Brommer, 2011), but not in bill size. Together, these studies suggest that birds can respond to changes in climate with potential implications for morphological and microevolutionary change.

Much of the environment which species inhabit is currently exhibiting rapid environmental change both in climate and in land use change. Modern climate change is occurring over a rapidly shifting landscape, and many regions have experienced widespread changes in urbanization (Arnfeld, 2003; McKinney, 2006). Several bird species are considered synanthropes that rely on bird feeders as a source of supplemental food and, in some cases, can survive and thrive in urban areas (Bosse et al., 2017; Marzluff, 2014). For example, northern cardinals reach higher abundances in urban areas than in rural areas due to warmer winter temperatures and supplemental food resources (Leston & Rodewald, 2006). Seminal studies documenting a relationship between seed size and Galapagos finches’ bill size demonstrated the importance of resource availability on bird morphology as seasons associated with large, tough seeds selected for birds with larger bills, while seasons with smaller seeds selected for smaller bills (Boag & Grant, 1981, 1984). With the onset of bird feeding as a popular pastime in many urban areas, supplemental seed may influence bill morphology (Robb, McDonald, Chamberlain, & Bearhop, 2008). For example, a study conducted on house finches (*Carpodacus mexicanus*) in the Sonoran Desert found that birds living in urban areas had larger bills with larger bite forces, likely a product of habitat‐specific selection pressures (Badyaev, Young, Oh, & Addison, 2008). Genomic regions under differential selection contain candidate genes for bill morphology across populations of great tits (*Parus major*), with birds in the United Kingdom evolving longer bills in response to supplementary feeding (Bosse et al., 2017). Given the increasing prevalence of urban areas (McKinney, 2006), it is likely that the characteristics of urban areas (e.g., urban heat islands, supplemental food) may complicate the predictions of biogeographic principles such as Allen's rule (Faurby & Araújo, 2016).

The goal of our study was to explore spatiotemporal changes in the bill size of northern cardinals (*Cardinalis cardinalis cardinalis*), a wide‐ranging, non‐migratory, synanthropic species that has been expanding its range northward over the past century (Dow & Scott, 1970; Halkin & Linville, 1999). The northward range expansion of cardinals has been associated with increases in winter minimum temperatures that may relax thermoregulatory constraints for populations occurring in northerly latitudes (La Sorte & Thompson, 2007; Root, 1988a; Zuckerberg, Woods, & Porter, 2009; Zuckerberg et al., 2011) and shifting resources due to the rise in popularity of bird feeders in northerly areas (Morneau et al., 1999). To test the predictions of Allen's rule, we measured bills from northern cardinal museum specimens collected across North America. We hypothesized that cardinal bill sizes would (1) be larger in warmer and more arid climates, (2) increase in regions characterized by warming temperatures, (3) be larger in more urban areas, and (4) increase in areas characterized by increasing housing density. By testing these predictions across a continental scale, we provide a novel examination of Allen's rule during a period of rapid environmental change.

## 2 MATERIALS AND METHODS

### 2.1 Specimen measurement

**Figure 1:**
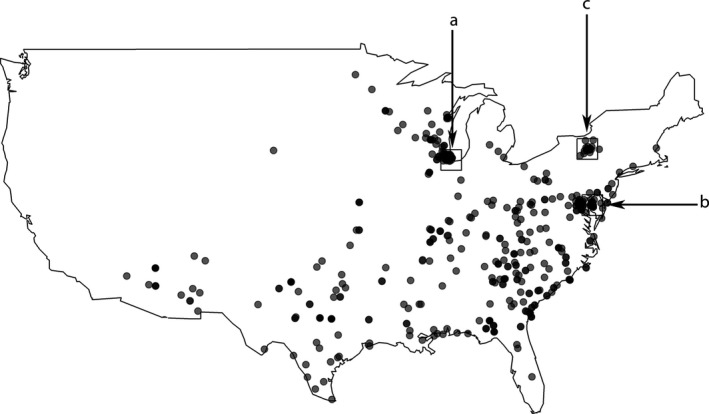
Map of collection locations of the 605 northern cardinal specimens used in this study indicated by gray circles. Black boxes represent the three locations at which temporal analyses were conducted: Chicago, Illinois (a); Washington, D.C. (b); and Ithaca, New York (c)

To quantify bill size of northern cardinals throughout their range, we took measurements of 605 museum specimens (Figure 1; Table S1). We used adult specimens that were collected in the wild and included information on collection locality and date. We took all measurements using Fowler digital calipers with 0.02 mm precision (Fowler Xtra‐Value Cal Electronic Calipers Model No. 54‐101‐150‐2). Only specimens of the Linnaean subspecies *Cardinalis cardinalis cardinalis* were included in this analysis. This subspecies occupies a broad range across eastern North America, which has expanded northward over time, while retaining stable genetic diversity (Smith et al., 2011).

We assumed a circular elliptical cone could approximate the shape of the bill and estimated bill surface area using the formula for the lateral surface area of a cone:

$$\left(\frac{W+D}{4}\right)\times L\times \pi$$

where *L* is the bill length measured from the proximal side of the nares to the bill tip; *W* is the bill width measured at the proximal side of the nares; and *D* is the depth of the bill measured at the point of the proximal side of the nares (Danner & Greenberg, 2015). Here, we use the term “proximal” in reference to the center of mass, where the proximal side of the bill is the side closest to the head and torso. We avoided dependence on facial plumage presence using the nares as a measurement guide. We measured each specimen three times and used the average of those measurements to quantify bill surface area. To avoid observer bias, a single observer (C.R.M.) measured all specimens. We measured tarsus length of each specimen as a proxy for body size in our analyses (Andrew, Awasthy, Griffith, Nakagawa, & Griffith, 2017; Danner et al., 2014; Labocha & Hayes, 2012; Teplitsky, Mills, Alho, Yarral, & Merilä, 2008). Bill measurements relative to the nares and skeletal measurements are considered robust in museum specimens; therefore, we assumed that our bill measurements from museum specimens reflected the measurements of a live specimen (Field, Lynner, Brown, & Darroch, 2013; Wilson & McCracken, 2008). Although tarsus length has been shown to vary according to Allen's rule in other species (Nudds & Oswald, 2007; Symonds & Tattersall, 2010), we considered tarsus length an appropriate proxy of passerine body size that has been used in previous studies and which positively correlates with skeletal body size (Senar & Pascual, 1997; Jawor et al. 2004; Bosse et al., 2017; Danner et al., 2017; Andrew et al., 2017).

### 2.2 Climate and housing density data

We obtained climate data collected in the United States between 1924 and 2016 using the R package “RFc” (Grechka et al., 2016). We extracted our climate data from the CRU CL 2.0, CESM1‐BGC CMIP5 rcp85, and WorldClim 1.4 current datasets (Arora et al., 2013; Hijmans, Cameron, Parra, Jones, & Jarvis, 2005; New, Lister, Hulme, & Makin, 2002). Climate variables represent monthly averages at each location. All climatic variables were z‐standardized to allow for easier interpretation of the coefficient estimates.

We calculated average air maximum and minimum temperature and relative humidity data for 30 years prior to collection at each location. These data represented the climate normal of a given geographical area. We also calculated climate variables for 4 years prior to specimen collection date at each location to represent the approximate generation time of northern cardinals. This approximate generation time was derived from $a+\frac{s}{1-s}$ *,* where *a* is age at normal breeding maturity for females and *s* is survival rate (Saether et al., 2005). Age at maturation for females was 1 year (Halkin & Linville, 1999; Richmond, 1978). We used an adult survival rate estimated by the Monitoring Avian Productivity and Survivorship (MAPS) program using transient Cormack–Jolly–Seber models (Desante, Kaschube, & Saracco, 2015; MAPS 2016).

We used housing density data for the United States between 1940 and 2016 obtained from US census data aggregated at the partial block level (Hammer, Stewart, Winkler, Radeloff, & Voss, 2004). Partial blocks are mapping units intermediate in scale between census block groups and block groups. As these data are a product of the US census, they provide decadal estimates of housing density. Therefore, we created 30‐year normal of housing density to match the climate data. Housing density served as a proxy both for the urban heat island microclimate and for the presence of supplemental seed. We used mean housing density estimated at a given specimen's coordinate.

### 2.3 Analysis

As bill surface area often co‐varies with body size in birds (Field et al., 2013), we took the residuals of bill size regressed on tarsus length as a measure of corrected bill size. Given concerns about the use of residuals (Freckleton, 2002), we also included tarsus as a predictor in all models (Tables S2 and S3). Positive residuals indicate a larger bill size relative to body size, while negative residuals indicate bill size proportionately smaller than body size. Northern cardinals express sexual dimorphism not only in body size, but also in corrected bill surface area. Males have larger bills than females throughout their range (Figure S1) and may experience higher exposure to air temperature and humidity variation as a result of singing more compared to females (Conner, Anderson, & Dickson, 1986) and increased respiratory water loss (Greenberg & Danner, 2012; Greenberg, Cadena et al., 2012). Therefore, we investigated the sexes separately for all analyses.

We used linear mixed models (LMM) using a Gaussian error distribution to assess the variation in bill surface area across range‐wide gradients of temperature and relative humidity for all birds and each sex separately. We ran two sets of models: one set for average minimum temperature and one set for average maximum temperature. We first aggregated our raw data by sex and collection location. We weighted cardinal bill surface area by the number of birds measured at each location to account for differences in the precision of estimates at each location and included an exponential correlation structure based on geographic coordinates accommodating spatial model inference (*N* = 345). We included the fixed effects of year, temperature, housing density, and humidity. Temperature, housing density, and humidity were all taken relative to their 30‐year average. We also included an interaction between temperature and humidity and an interaction between temperature and housing density. We incorporated observation ID as a random effect to serve as a grouping factor for the spatial correlation matrix. We ran models using the R package “nlme” (Pinheiro, Bates, DebRoy, & Sarkar, 2011). We used an alpha level of 0.05 for significant results.

For purposes of visualization, we used geographically weighted regression (GWR) to allow modeled relationships between climate and bill surface area to vary across geographic regions (Charlton & Fotheringham, 2009). GWR is primarily an exploratory approach, which allows local and regional non‐stationarity in parameter coefficients. We included two separate relationships in this analysis, investigating the relationship between bill surface area and relative humidity, and bill surface area and relative humidity x average minimum temperature for all birds. We used fixed bandwidths (selected using AIC_*c*_ in R package “spgwr”) to inform spatial weighting of each data point (Bivand & Yu, 2008) and estimated local coefficients using a Gaussian kernel function in the R package “spgwr” (Bivand & Yu, 2008).

Due to a sparsity of samples during the full 85‐year period over all regions within our study range, we investigated temporal variation of bill surface area in three separate subregions that had adequate coverage across the 85‐year time span: Chicago, IL, Ithaca, NY, and Washington, D.C. (Figure 1). The northern cardinal dispersal distance is conservatively estimated at 10 km; therefore, we used a 10 km radius distance for each location in this subregion analysis (Ausprey & Rodewald, 2013; Donovan & Flather, 2002). Using general linear models, we tested the relationship between bill surface area and year, bill surface area and housing density, and bill surface area and minimum temperature for three groups: females, males, and all birds. We used decadal estimates for housing density in this temporal analysis. *R* ^2^ values for our generalized mixed models and simple linear models were calculated using the package “MuMIn” in R (Barton, 2018). We z‐standardized all independent variables to allow straightforward comparison and interpretation of the coefficient estimates and conducted all analyses in R Version 3.3.1 (R Core Team 2016).

## 3 RESULTS

### 3.1 Geographic differences in bill size

**Figure 2:**
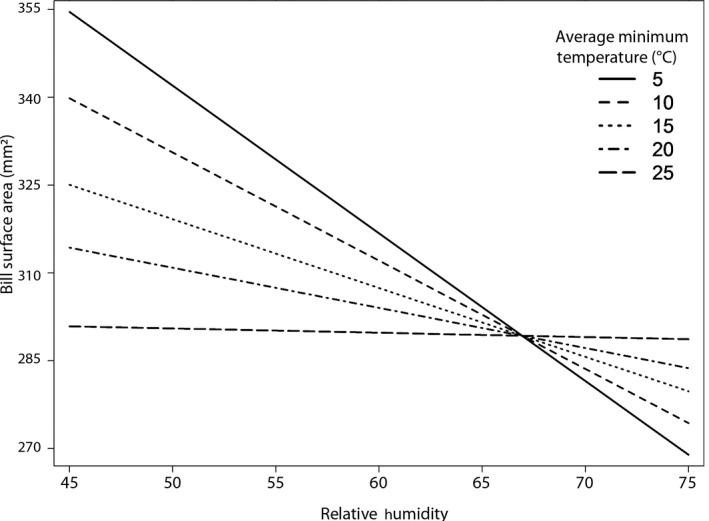
Interaction between average minimum temperature and relative humidity effect on bill surface area of all birds. In cooler regions, bill surface area decreased with increasing humidity. In warmer regions, relative humidity had less effect on bill surface area. Each line represents a different average minimum temperature, while the *x*‐axis represents relative humidity

While we conducted analyses for both average minimum and maximum temperatures (Table S4), we present results for minimum temperature because minimum temperature and maximum temperature values were highly correlated (*r* = .96). We selected minimum temperature because the cardinal range has shifted north over the past century (Halkin & Linville, 1999; La Sorte & Thompson, 2007) and is thought to be related to warming minimum temperatures. We hypothesized that minimum temperatures would play a larger role in cardinal ecology as their northerly range boundary, and hence expansion, is correlated with isotherms of minimum temperature (Root, 1988b). Cardinals had smaller bills in more arid regions (β = −8.66, *SE* = 1.91, *p* = .00), with most of this pattern being exhibited by male as opposed to female birds (Table 1). This relationship was modified by average minimum temperature, with birds residing in warmer areas having a weaker relationship with relative humidity than cardinals residing in cooler areas (β = 4.08, *SE* = 1.92, *p* = .03) (Figure 2). Contrary to our predictions, cardinals found in areas with higher housing density had smaller bill surface areas (β = −3.62, *SE* = 1.44, *p* = .01). We found no significant temporal trends for all birds (β = 0.56, *SE* = 1.66, *p* = .75), females (β = 0.03, *SE* = 0.10, *p* = 0.75), or males (β = −0.01, *SE* = 0.07, *p* = .89) across the entire range (Table 1). Using tarsus as a predictor did not change our results (Table S2). The conditional *R* ^2^ was 0.069 for the “all birds” model, 0.038 for the “female‐only” model, and 0.117 for the “male‐only” model.

**Table 1:** Results of the model of bill surface area across the United States for female and male northern cardinals

Variable	Estimate	Standard error	*p*‐value
All birds			
Sex	15.644	4.183	<.001
Year	0.555	1.618	.732
Mintemp	3.317	1.748	.059
Hum	−8.314	1.868	<.001
Hden	−3.507	1.404	.013
Mintemp × Hum	3.240	1.891	.088
Mintemp × Hden	−1.876	1.156	.106
Females			
Year	0.033	0.101	.745
Mintemp	4.396	3.256	.180
Hum	−1.713	4.735	.718
Hden	−3.101	2.310	.182
Mintemp × Hum	1.059	3.554	.766
Mintemp × Hden	0.144	3.586	.968
Male			
Year	−0.009	0.070	.893
Mintemp	4.198	2.087	.045
Hum	−10.075	2.024	<.001
Hden	−3.017	1.729	.082
Mintemp × Hum	0.982	2.482	.693
Mintemp × Hden	−1.816	1.278	.157

Predictor variables include sex, year, average minimum temperature (mintemp), relative humidity (hum), and housing density (hden), against which bill size was regressed. We present the parameter estimate, standard error of that estimate, and the *p*‐value.

**Figure 3:**
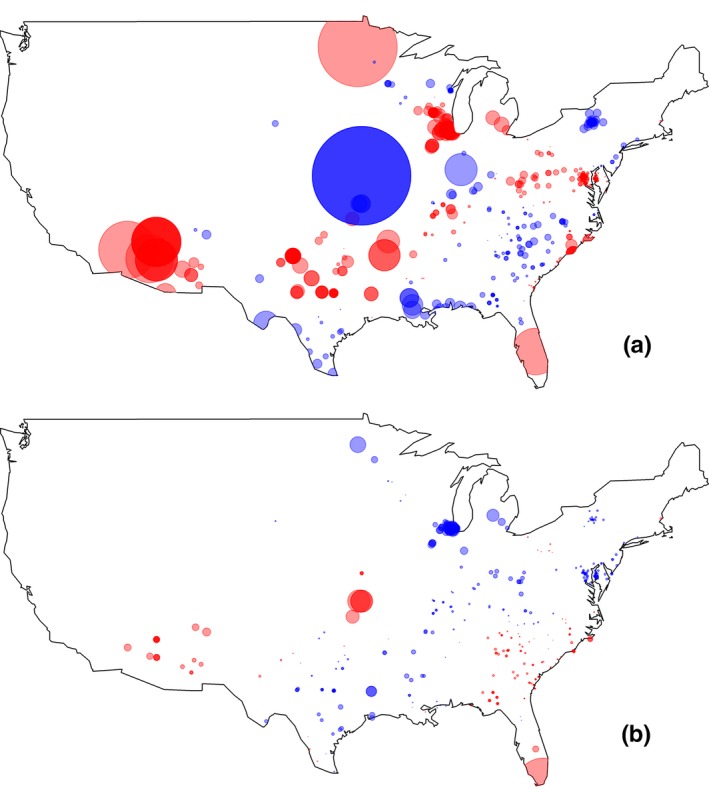
Maps of the resulting coefficients from the geographically weighted regression analysis of (a) bill size as a function of relative humidity, and (b) bill size as a function of average minimum temperature × relative humidity for all birds. Each circle represents the statistical relationship between bill size and the independent climate variable estimated for an individual point or bird. Red circles represent positive coefficients, and blue circles represent negative coefficients

We conducted two separate GWR analyses to explore the geographic relationships resulting from our range‐wide mixed effect model set. We used a fixed bandwidth of 144 km when testing the relationship between bill surface area and relative humidity for all birds. The relationship between bill surface area and relative humidity varied across the United States. Birds found in the far southwestern, southeastern, and northeastern United States and the Midwest commonly had larger bill surface areas in more humid conditions (Figure 3a). However, many birds residing in areas from Texas to the mid‐Atlantic United States had smaller bill surface areas in more humid conditions exemplifying the geographically complex response in bill size related to climatic factors (Figure 3a).

We used a fixed bandwidth of 294 km for all birds when assessing the relationship between bill surface area and the interaction between relative humidity and average minimum temperature. Birds living in the far southwest and lower southeast of the United States exhibited a strong positive interaction between relative humidity and average minimum temperature: Bill surface area increased with increasing relative humidity, a relationship augmented by higher temperatures (Figure 3b). These patterns agree with those found in our linear mixed models, in which the positive relationship between bill surface area and relative humidity across the range was also found to be augmented by high minimum temperatures (Figure 2). The direction of this interaction varied across the United States, with birds residing in areas ranging from Texas to the northeastern United States exhibiting a negative interaction.

### 3.2 Changes in bill size over time

**Figure 4:**
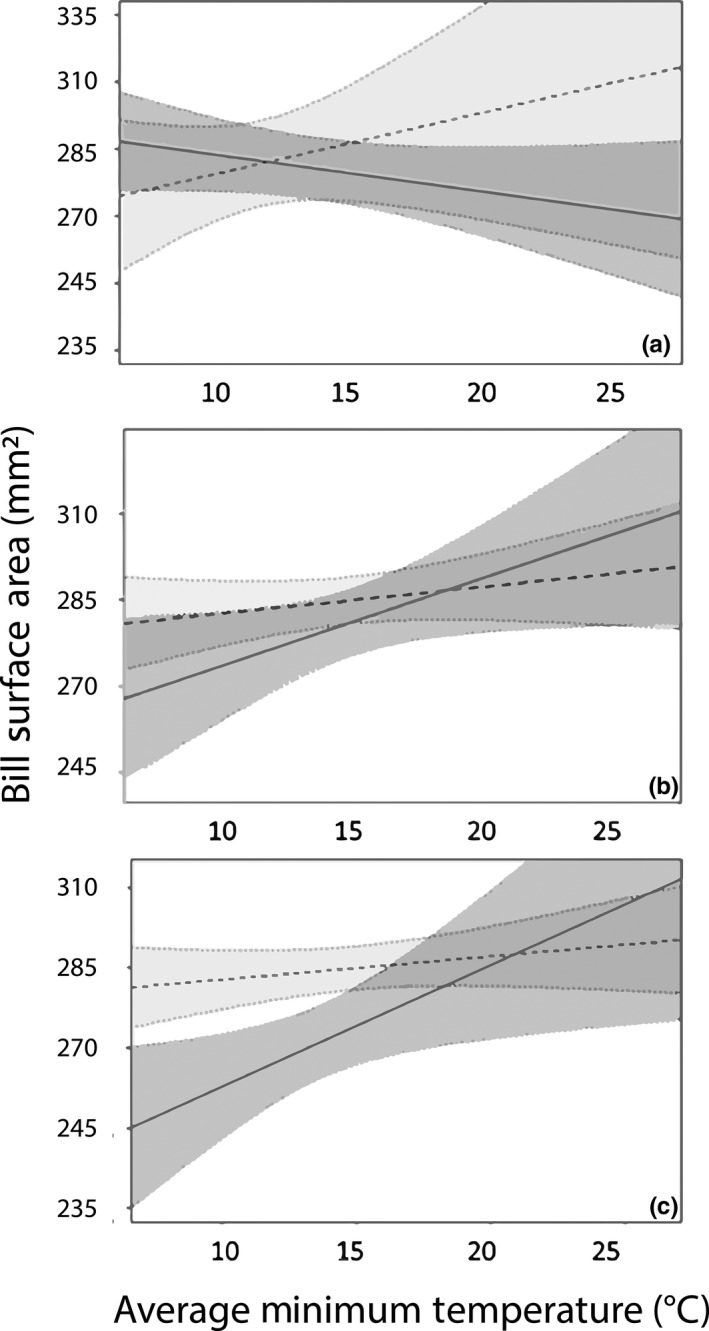
The relationship between male and female bill size and average minimum temperature in Chicago, Illinois (a), Washington, D.C. (b), and Ithaca, New York (graph c). Solid lines and dark gray polygons represent female trends; dashed lines and light gray polygons represent male trends; and polygons represent 95% confidence intervals. Female bill size increased with average minimum temperature in Ithaca and Washington, D.C., but not in Chicago. Male bill size did not vary with temperature

**Figure 5:**
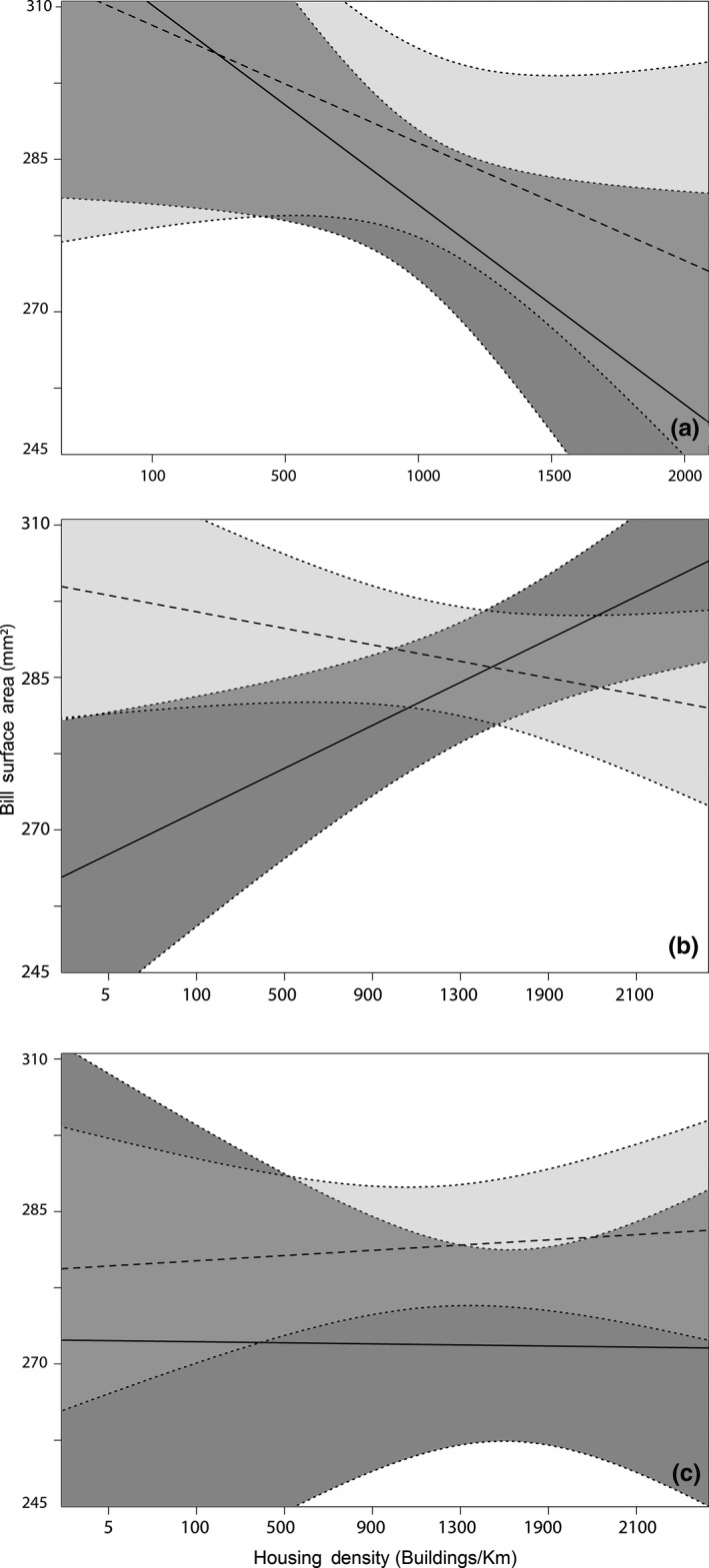
The relationship between male and female bill size and housing density in Chicago, Illinois (a), Washington, D.C. (b), and Ithaca, New York (c). Solid lines and dark gray polygons represent female trends, dashed lines and light gray polygons represent male trends; and polygons represent 95% confidence intervals. Female bill size increased with housing density in Washington, D.C., but decreased with housing density in Chicago. Bill size did not change with housing density in Ithaca

Within the Washington, D.C., area, we found that bill surface area of all birds (*N* = 66) increased with rising minimum temperatures (*R* ^2^ = .06), a pattern found in females but absent in males (Table 2; Figure 4a). Female bill surface area increased with increasing housing density in the Washington, D.C., area (*R* ^2^ = .34) (Figure 5a). In Chicago, no birds (*N* = 29) exhibited relationships with either minimum temperature or with year, but female bill size decreased with increasing housing density (*R* ^2^ = .52) (Table 2; Figure 5b). In Ithaca, NY, the bills of female cardinals (*N* = 34) increased significantly with increases in minimum temperature (*R* ^2^ = .59). In contrast, male bill surface area did not change over gradients of minimum temperature (Table 2; Figure 4c). Bill surface areas did not change over a gradient of housing density in the Ithaca area (Figure 5c).

**Table 2:** Model results for female and male northern cardinals over time

Location	Model^a^	Estimate	Standard error	*p*‐value
Washington				
All Birds				
	Mintemp	4.335	2.205	.054
	Year	−0.272	0.349	.438
	H. Density	0.011	0.014	.409
Females				
	Mintemp	9.639	4.479	.044
	Year	0.265	0.791	.742
	H. Density	0.056	0.019	.009
Males				
	Mintemp	2.933	2.498	.247
	Year	−0.388	0.387	.322
	H. Density	−0.021	0.017	.224
Chicago				
All Birds				
	Mintemp	−4.462	3.823	.253
	Year	0.220	0.258	.402
	H. Density	−8.775	3.874	.035
Females				
	Mintemp	−5.235	3.285	.146
	Year	0.234	0.281	.427
	H. Density	−13.098	−2.545	.044
Males				
	Mintemp	8.693	7.934	.289
	Year	0.348	0.465	.465
	H. Density	−7.721	5.219	.167
Ithaca				
All Birds				
	Mintemp	1.984	2.726	.472
	Year	0.021	0.142	.148
	H. Density	0.011	0.014	.409
Females				
	Mintemp	15.151	5.169	.026
	Year	−0.459	0.197	.059
	H. Density	−0.279	5.622	.962
Males				
	Mintemp	2.259	3.780	.556
	Year	0.162	0.162	.327
	H. Density	1.373	3.648	.713

We present results for each subregion and included predictors of average minimum temperature (mintemp), housing density (hden), and year. Female bill size in Ithaca, New York, and Washington, D.C., increased with increasing mintemp. No temporal relationships are evident for male bill size. Model descriptors include the parameter estimate, standard error of that estimate, and the *p*‐value.

a Minimum temperature, housing density, and year were included in separate linear models.

## 4 DISCUSSION

Species and populations coping with a rapidly changing climate are capable of significant microevolutionary adjustments ranging from shrinking body sizes (Caruso, Sears, Adams, & Lips, 2014) to reductions in melanism (De Jong & Brakefield, 1998). In this study, we provided a unique biogeographic test of Allen's rule during a period of rapid environmental change. Our findings support past evidence of larger bills in drier environments (Campbell‐Tennant et al., 2015; Danner et al., 2017). Over an 85‐year period, cardinal bill size increased with warming temperatures in two of three geographic subregions, but only for females. Additionally, the relationship between bill size and housing density was complex. Females inhabiting highly developed areas of Chicago had smaller bills than those inhabiting the periphery of the city, while females in developed areas of Washington, D.C., had larger bill sizes.

Northern cardinals inhabit a broad swath of North America and must adapt to a wide range of temperature and aridity. We found strong support that cardinals occupying drier regions had bigger bills, but this pattern was strongest in males. These results suggest that bill size is a flexible trait important for water conservation and represents a climate–morphological relationship that varies between the sexes (Danner et al., 2017; Greenberg, Cadena et al., 2012). A possible reason for this sex‐based difference is that males sing for extensive periods, often on exposed branches, expending energy and water while maintaining territories during breeding (Conner et al., 1986; Richmond, 1978; Vondrasek, 2006). This behavior could invoke thermoregulatory costs due to increased exposure to ambient humidity and temperature. Activities such as singing can lead to increased respiratory water loss, and birds with larger bills can potentially offset these costs by emitting dry heat (Greenberg, Cadena et al., 2012). Our geographic analysis suggests pronounced differences in the strength of the relationship between bill size and temperature in arid regions (Figure 3), providing additional support that bills are a key feature for heat exchange and water control (Danner et al., 2017; Noakes, Wolf, & McKechnie, 2016; Smith, O'Neill, Gerson, & Wolf, 2015; Van de Ven et al., 2016).

Cardinals inhabiting warmer climates had a weaker relationship with relative humidity than those living in cooler climates, although this relationship varied over space (Figures 2 and 3). Previous work on the effects of these two climate factors on bill size supports these results. The internal conchae structures of bird bills are critical for mediating water evaporation and are significantly larger in song sparrow subspecies occupying arid regions (Danner et al., 2017; Luther & Danner, 2016). A study conducted on Australian passerines found that the positive interaction of humidity and maximum temperature promoted larger bill sizes (Gardner et al., 2016). We found a geographically complex relationship between bill size and humidity that varies from the northeastern United States to the southwestern United States. In areas characterized by high temperatures, such as the southeastern United States, bill sizes were relatively large in areas with high humidity, whereas in cooler areas such as the northeastern United States, the interaction between humidity and temperature was weak (Figure 3). While we predicted cardinals would have larger bills in arid environments to mitigate evaporative water loss, selection pressures may also exist in regions of high humidity to maximize dry heat exchange because evaporative cooling is less efficient in saturated environments (Powers, 1992). With predicted shifts in precipitation and humidity across the southern United States (Easterling et al., 2000; IPCC, 2014), climate change may cause variable shifts in bill sizes throughout the northern cardinal range.

We predicted that increasing temperatures over time would promote larger bill sizes for resident populations in our three subregions. Bill sizes did not demonstrate significant changes over time, but female cardinals exhibited a strong positive relationship with increasing temperatures in two subregions. Female bill size increased at a rate of 9 mm^2^/°C in Washington, D.C., and 15 mm^2^/°C in Ithaca (Figure 4). Microevolutionary changes can occur over relatively short periods of time (e.g., decades) (De Jong & Brakefield, 1998; Réale et al. 2003) and at different rates between sexes (Evans & Gustafsson, 2017). Changes in morphological features between sexes may be indicative of sexual selection. A study conducted in Gotland, Sweden, found a decrease in the white‐feathered forehead patch size of male collared flycatchers (*Ficedula albicollis*) due to lessened breeding site density and competition due to warming spring temperatures (Evans & Gustafsson, 2017). Although our findings point to the importance of sexual dimorphism in climate–morphological relationships, future research is needed to further identify the mechanisms underlying these sex‐specific relationships. We may be underestimating the relationships presented between bill size and climate per Allen's rule because we used tarsus length as our proxy to control for skeletal body size. It is possible that tarsus length in northern cardinals may follow Allen's rule (Nudds & Oswald, 2007; Symonds & Tattersall, 2010) or be confounded by other natural history characteristics such as foraging behavior (Aldrich 1984). Additionally, the limited sample sizes available in the temporal analysis may have influenced our ability to identify significant relationships between bill size and environmental parameters. Consequently, although we found support for increasing bill sizes associated with warming temperatures, future longitudinal studies are needed to explore the consistency of these relationships for different species and regions.

The complexity of the relationship between climate and bill size may result from competing influences of urbanization which may act to dampen biogeographic processes (Faurby & Araújo, 2016). The northern cardinal is a synanthropic species commonly found in urban and suburban areas (Leston & Rodewald, 2006; Rodewald & Shustack, 2008). With increased use of urban areas and supplemental food, resource availability may be as important a driver of bill size variability as climate. Cardinals are not considered diet specialists, but feed on multiple resources including seed, fruit, and insects (Wilman et al., 2014), which are available across their range. Seminal work on the role of seed size availability and changes in bill size in Galapagos finches clearly exhibit the relationship between resource availability and natural selection (Boag & Grant, 1981, 1984; Grant & Grant, 2002), but these relationships were ultimately mediated by climate and extreme weather events (e.g., drought) (Grant et al., 2017).

With the increasing popularity of backyard bird feeding (Robb et al., 2008) and the urban heat island effect, we predicted bill size to increase with housing density. This relationship was not supported over geographic scales, and although we found that female bill size increased with increasing housing density in Washington, D.C., bill size decreased significantly with housing density in Chicago, and we found no relationship with in Ithaca, NY Potential reasons for this pattern are that housing density may not be an adequate proxy for the prevalence of bird feeders and there may be additional aspects of the urban environment (e.g., noise and light pollution) that may influence selection pressures on bill size. Variation in housing density captures strong and complex environmental changes across urban to rural land use gradients, and it is possible that supplemental feeding is most prominent in areas with lower housing density. This may lead to a negative relationship between bill size and housing density should supplementary feeding be more common in suburban and rural areas as opposed to highly urban areas. Alternatively, urban areas are warmer due to the UHI, and as per Allen's rule, it is possible that these warmer conditions promote larger bills. Although cardinal bill sizes had a positive relationship with housing density in Washington, D.C., we found the opposite pattern for Chicago. It is possible that the contrasting results found for Chicago were due to differences in local climatology or higher frequency of cold extremes in Chicago that could counteract the warming effects of the UHI. We chose these cities as large metropolitan areas with well‐established northern Cardinal populations and an adequate sample size of museum specimens over time. The differences between the three cities point to the complexity of the relationship between morphological adaptations and rapidly urbanizing environments. Further research will be necessary to better understand how anthropogenic change influences species in an increasingly urbanized world.

Our study elucidates the complexity and challenges of exploring the predictions of biogeographic rules, such as Allen's rule, during a time of rapid environmental change. Cardinal bill sizes were larger in more arid environments and generally increased with warming temperatures. These relationships, however, were complicated by sex‐specific differences and the role of urbanization. As human‐induced climate and land use change continue, northern cardinals and other bird species will experience novel conditions. Northern cardinal ranges will likely continue to expand northward and occupy novel urban areas where unique selection pressures will influence morphology over time (Scheffers et al., 2016). While climate and land use change are often described as extinction drivers (Jetz, Wilcove, & Dobson, 2007; Møller, Rubolini, & Lehikoinen, 2008; Selwood, McGeoch, & Mac Nally, 2014), species may respond to anthropogenic drivers through microevolutionary changes in line with the predictions of biogeographical principles such as Allen's rule.

## AUTHOR CONTRIBUTIONS

C.R.M., C.E.L., and B.Z. designed research; C.R.M. collected museum specimen data; C.R.M. and C.E.L. analyzed the data with guidance from B.Z.; C.R.M, C.E.L, and B.Z. wrote the manuscript.
