# Supplementary material for: Bill size variation in northern cardinals associated with anthropogenic drivers across North America
Source: Ecol Evol. 2018 Apr 17;8(10):4841–51. doi: 10.1002/ece3.4038 (PMC5980444; doi:10.1002/ece3.4038)

**Appendix I**

**Appendix Tables and Figures:**

**Table S1:** Appendix of museum specimens that were used in this study arranged by collection ID number. Measurements include bill surface area (mm^2^) approximated from length, width and depth of specimens’ bills and tarsus length (mm). Locality represents the locality at which the specimen was collected. Museums which house the specimens currently are the Angelo State Natural History Collection (ASNHC), Cornell University Museum of Vertebrates (CUMV), Delaware Museum of Natural History (DMNH), Denver Museum of Nature and Science (DMNS), Field Museum of Natural History (FMNH), Harvard Museum of Comparative Zoology (MCZ), Moore Lab of Zoology (MLZ), Museum of Southwestern Biology (MSB), National Museum of Natural History (NMNH), University of Washington Burke Museum of Natural History (UWBM), and the University of Wisconsin Zoological Museum (UWZM).

| **Museum ID** | **Collection Number** | **Bill Surface Area** | **Tarsus** | **Locality** |
| --- | --- | --- | --- | --- |
| ASNHC | 48 | 273.047 | 12.657 | USA; Texas; Tom Green County; Nasworthy |
| ASNHC | 273 | 268.908 | 15.423 | USA; Texas; Tom Green County; San Angelo |
| ASNHC | 306 | 284.794 | 13.56 | USA; Texas; Tom Green County; Nasworthy |
| ASNHC | 1150 | 264.676 | 13.237 | USA; Texas; Callahan County; Admiral |
| ASNHC | 1151 | 248.407 | 22.267 | USA; Texas; Callahan County; Admiral |
| UAM | 1611 | 312.54 | 22.45 | USA; Minnesota; Ramsey County; St. Paul |
| WFVZ | 2566 | 289.552 | 24.8 | USA; Arizona; Maricopa County; Wickenberg |
| UWZM | 7365 | 243.082 | 22.207 | USA; Georgia; Bulloch County; Statesboro |
| UAM | 9047 | 268.251 | 21.157 | USA; Minnesota; Ramsey County; St. Paul |
| CUMV | 9669 | 320.346 | 21.457 | Mexico; Hidalgo County; Jacala |
| DMNH | 9988 | 270.656 | 23.63 | USA; New Mexico; Catrow County; Glenwood |
| CUMV | 11222 | 327.181 | 25.183 | USA; Arizona; Pima County; Menager's Dam |
| CUMV | 11543 | 291.191 | 21.573 | USA; Louisiana; East Baton Rouge Parish; Baton Rouge |
| CUMV | 11762 | 312.875 | 21.493 | Mexico; Nuevo Leon County; Monterrey |
| CUMV | 11763 | 260.73 | 21.52 | Mexico; Nuevo Leon County; Monterrey |
| CUMV | 11764 | 311.99 | 23.173 | Mexico; Nuevo Leon County; Monterrey |
| CUMV | 11771 | 247.655 | 21.85 | USA; Texas; Cameron County; Los Fresnos |
| CUMV | 11797 | 281.706 | 22.947 | Mexico; Yucatan County; Progreso |
| UWZM | 13930 | 265.435 | 23.593 | USA; Wisconsin; Dane County; Madison |
| UWZM | 14175 | 322.186 | 23.637 | USA; Wisconsin; Dane County; Madison |
| UWZM | 14608 | 307.447 | 25.093 | USA; Wisconsin; Marathon County; Wausau |
| UWZM | 14781 | 278.952 | 22.363 | USA; Wisconsin; Dodge County; Horicon Marsh |
| UWZM | 14823 | 249.972 | 22.533 | USA; Wisconsin; Dane County; Mazomanie |
| UWZM | 16011 | 297.587 | 22.147 | USA; Wisconsin; Wauschana County; Wautoma |
| UWZM | 16235 | 301.848 | 22.933 | USA; Wisconsin; Dane County; Madison |
| UWZM | 16236 | 283.207 | 21.277 | USA; Wisconsin; Dane County; Madison |
| UWZM | 17702 | 281.545 | 21.307 | USA; Wisconsin; Dane County; Madison |
| MSB | 18505 | 375.781 | 25.983 | USA; New Mexico; Grant County; Red Rock Wildlife Management Area |
| UAM | 19181 | 288.703 | 24.987 | USA; New York; Cayuga County; Montezuma |
| CUMV | 21102 | 277.661 | 23.26 | USA; New York; Tompkins County; Ithaca |
| CUMV | 21896 | 337.94 | 23.59 | USA; Ohio; Carroll County; Amsterdam |
| CUMV | 21957 | 268.491 | 22.173 | USA; New York; Tompkins County; Ithaca |
| DMNH | 22073 | 312.601 | 24.1 | USA; New Mexico; Grant County; Red Rock Wildlife Management Area |
| DMNS | 24081 | 257.184 | 22.653 | USA; Texas; Maverick County; Quemado |
| MSB | 24191 | 327.703 | 23.863 | USA; Texas; Chambers County; High Island |
| MSB | 24193 | 325.213 | 23.747 | USA; Texas; Starr County; San Romana |
| MSB | 24246 | 322.454 | 22.95 | USA; Texas; Chambers County; High Island |
| CUMV | 24349 | 257.876 | 23.457 | USA; New York; Tompkins County; Enfield |
| MSB | 24474 | 287.406 | 22.567 | USA; New Mexico; Eddy County; Carlsbad |
| MSB | 26785 | 288.25 | 24.45 | USA; Louisiana; Iberville Parish; St. Gabriel |
| DMNH | 26914 | 281.326 | 24.64 | USA; Arizona; Yuma County; Parker |
| MSB | 29264 | 338.882 | 23.22 | USA; Texas; Refugio County; Tivoli |
| MSB | 29888 | 278.762 | 23.317 | USA; Florida; Alacua County; Gainesville |
| MSB | 30862 | 264.55 | 24.535 | USA; New Jersey; Cape May County; Cape May West |
| CUMV | 31144 | 374.923 | 22.783 | Mexico; Veracruz County; Sontecomapan |
| CUMV | 34599 | 323.765 | 24.897 | Mexico; Campeche County; Sabancuy |
| CUMV | 34600 | 317.609 | 22.94 | Mexico; Yucatan County; Chicxulub Puerto |
| CUMV | 34601 | 336.338 | 24.203 | Mexico; Quintana Roo County; Chetumal |
| CUMV | 34603 | 307.38 | 21.155 | Mexico; Quintana Roo County; Chetumal |
| CUMV | 34604 | 301.448 | 22.857 | Mexico; Quintana Roo County; Chetumal |
| CUMV | 34605 | 291.948 | 23.437 | Mexico; Campeche County; Sabancuy |
| CUMV | 35102 | 253.327 | 22.75 | USA; New York; Westchester County; Yonkers |
| CUMV | 37456 | 266.608 | 24.27 | USA; New York; Tompkins County; Lansing |
| CUMV | 37457 | 299.394 | 21.083 | USA; New York; Tompkins County; Ithaca |
| DMNS | 38687 | 239.057 | 24.063 | USA; Texas; Dimmit County; Catarina |
| MSB | 40218 | 332.284 | 23.717 | USA; New Mexico; Grant County; Gila River |
| MSB | 41391 | 386.216 | 23.75 | USA; New Mexico; Hidalgo County; Little Hatchet Mountains |
| CUMV | 43832 | 273.038 | 21.483 | USA; New York; Tompkins County; Ithaca |
| CUMV | 43863 | 256.33 | 23.513 | USA; New York; Tompkins County; Dryden |
| CUMV | 44162 | 248.425 | 19.887 | USA; New York; Tompkins County; Ithaca |
| DMNH | 45215 | 272.247 | 22.783 | USA; Arizona; Maricopa County; Scottsdale |
| DMNH | 45216 | 286.237 | 24.625 | USA; Arizona; Maricopa County; Scottsdale |
| DMNH | 45217 | 253.902 | 23.36 | USA; Texas; Victoria County; Victoria |
| CUMV | 48335 | 284.098 | 23.907 | USA; New York; Tompkins County |
| CUMV | 48338 | 289.203 | 21.873 | USA; New York; Cortland County; Harford |
| CUMV | 48342 | 269.99 | 22.5 | USA; New York; Cortland County |
| CUMV | 48344 | 288.553 | 23.31 | USA; New York; Tompkins County; Ithaca |
| CUMV | 48345 | 241.376 | 20.157 | USA; New York; Tompkins County; Dryden |
| DMNH | 48488 | 228.891 | 21.14 | USA; Texas; Goliad County; Berclair |
| DMNH | 48489 | 245.377 | 21.39 | USA; Texas; Armstrong County; Wayside |
| DMNH | 48491 | 260.664 | 23.32 | USA; Texas; Armstrong County; Wayside |
| DMNH | 48509 | 240.348 | 22.59 | USA; Georgia; Grady County; Sherwood Plantation |
| DMNH | 48510 | 226.327 | 22.423 | USA; Texas; Webb County; Laredo |
| CUMV | 49033 | 272.61 | 22.99 | USA; New York; Tompkins County; Ithaca |
| CUMV | 50497 | 248.519 | 22.823 | USA; New York; Tompkins County; Ithaca |
| CUMV | 50814 | 261.729 | 23.02 | USA; New York; Tompkins County; Trumansburg |
| CUMV | 51391 | 283.072 | 21.19 | USA; New York; Tompkins County; Ithaca |
| CUMV | 51398 | 266.385 | 23.08 | USA; New York; Tompkins County; Ithaca |
| CUMV | 51461 | 298.225 | 22.49 | USA; New York; Brooks County; Vestal |
| CUMV | 51595 | 299.069 | 20.423 | USA; New York; Shuyler County; Odessa |
| WFVZ | 51858 | 255.541 | 22.56 | USA; Texas; Brooks County; Falfurrian |
| WFVZ | 51860 | 228.768 | 22.523 | USA; Texas; Fringin County; Nobility |
| WFVZ | 51862 | 219.769 | 22.785 | USA; Texas; Kendall County; Schneider Ranch |
| WFVZ | 51863 | 256.758 | 24.157 | USA; Texas; Tarrant County; Fort Worth |
| WFVZ | 51866 | 237.245 | 24.327 | USA; Texas; Grayson County; Sherman |
| WFVZ | 51867 | 235.161 | 22.66 | USA; Texas; Denton County; Denton |
| WFVZ | 51868 | 243.947 | 22.81 | USA; Texas; Dallas County; Dallas |
| WFVZ | 51869 | 218.494 | 24.12 | USA; Texas; Dallas County; Dallas |
| CUMV | 51988 | 290.212 | 24.647 | USA; New York; Tompkins County; North Lansing |
| CUMV | 52514 | 276.548 | 21.313 | USA; New York; Tompkins County; Ithaca |
| CUMV | 52752 | 283.438 | 22.367 | USA; New York; Tompkins County; Ithaca |
| CUMV | 52865 | 251.074 | 19.877 | USA; New York; Steuben County; Lindley |
| CUMV | 53163 | 265.135 | 22.53 | USA; New York; Tompkins County; Ithaca |
| CUMV | 53174 | 253.462 | 21.25 | USA; New York; Tompkins County; Brooktondale |
| CUMV | 53196 | 295.403 | 21.123 | USA; New York; Tompkins County; Ithaca |
| CUMV | 53412 | 234.306 | 20.637 | USA; New York; Tioga County; Candor |
| CUMV | 54697 | 286.191 | 23.183 | USA; New York; Tompkins County; Ithaca |
| CUMV | 55012 | 276.293 | 21.69 | USA; New York; Tompkins County |
| CUMV | 55231 | 284.19 | 22.417 | USA; New York; Tompkins County |
| CUMV | 55473 | 273.251 | 24.283 | USA; New York; Chennung County; Horseheads |
| CUMV | 55475 | 255.205 | 20.743 | USA; New York; Tompkins County; Dryden |
| CUMV | 55501 | 283.239 | 21.583 | USA; New York; Tompkins County; Ithaca |
| CUMV | 55702 | 264.508 | 21.163 | USA; New York; Tompkins County; Ithaca |
| CUMV | 55997 | 258.448 | 21.873 | USA; New York; Tompkins County; Newfield |
| MLZ | 69140 | 201.938 | 24.383 | USA; North Carolina; Wake County; Raleigh |
| MLZ | 69141 | 224.783 | 25.553 | USA; North Carolina; Wake County; Blue Jay Point County Park |
| MLZ | 69142 | 230.089 | 25.49 | USA; North Carolina; Johnston County; Howell Woods |
| MLZ | 69143 | 226.473 | 25.573 | USA; North Carolina; Johnston County; Howell Woods |
| MLZ | 69144 | 223.527 | 25.153 | USA; North Carolina; Durham County |
| UWBM | 79884 | 319.661 | 23.06 | USA; Virginia; Loudoun County; Aldie |
| UWBM | 80125 | 327.621 | 21.553 | USA; Louisiana; St. Tammany Parish |
| UWBM | 82684 | 359.932 | 22.05 | Mexico; Baja California; San Antonio |
| UWBM | 86245 | 322.5 | 21.007 | USA; Texas; Bell County; Fort Hood Military Reservation |
| UWBM | 100165 | 367.64 | 22.447 | USA; Kansas; Labette County; Big Hill Wildlife Management Area |
| UWBM | 100166 | 361.044 | 23.367 | USA; Texas; Nacodoches County; Alazan Bayou Wildlife Management Area |
| UWBM | 100167 | 362.576 | 23.017 | USA; Texas; Brown County; Zephyr |
| UWBM | 100168 | 334.589 | 22.133 | USA; Kansas; Labette County; Big Hill Wildlife Management Area |
| UWBM | 100169 | 318.81 | 22.963 | USA; Lousiana; Tensas Parish; Buckhorn Wildlife Management Area |
| UWBM | 100170 | 292.777 | 22.777 | USA; Kansas; Montgomery County; Elk City Wildlife Management Area |
| UWBM | 100174 | 294.039 | 20.813 | USA; Texas; Brown County; Zephyr |
| UWBM | 100175 | 303.691 | 21.707 | USA; Texas; Brown County; Zephyr |
| UWBM | 100176 | 375.363 | 23.56 | USA; Louisiana; Ouachita Parish; Russell Sage Wildlife Management Area |
| UWBM | 100242 | 308.306 | 20.65 | USA; Texas; Brown County; Zephyr |
| UWBM | 100619 | 367.348 | 23.113 | USA; Arizona; Coconino County; Flagstaff |
| UWBM | 100620 | 399.186 | 23.623 | USA; Arizona; Coconino County; Flagstaff |
| UWBM | 100621 | 423.622 | 24.573 | USA; Arizona; Coconino County; Flagstaff |
| UWBM | 100622 | 367.587 | 22.547 | USA; Arizona; Coconino County; Flagstaff |
| UWBM | 100623 | 380.959 | 24.193 | USA; Arizona; Coconino County; Flagstaff |
| UWBM | 101028 | 327.268 | 21.413 | Mexico; Coahuila County; Municipio de Guerrero |
| UWBM | 101029 | 328.274 | 22.597 | Mexico; Coahuila County; Municipio de Guerrero |
| UWBM | 101030 | 284.524 | 22.693 | Mexico; Coanuila County; Santa Domingo |
| UWBM | 101132 | 301.07 | 20.173 | Mexico; Michoacan County; La Mira |
| UWBM | 101159 | 310.946 | 20.33 | Mexico; Michoacan County; La Mira |
| UWBM | 101160 | 277.649 | 19.46 | Mexico; Michoacan County; La Mira |
| UWBM | 101707 | 333.611 | 22.183 | USA; Texas; Coryell County; Jonesboro |
| UWBM | 103342 | 268.877 | 21.3 | USA; North Carolina; Alachua County; Gainesville |
| UWBM | 103345 | 334.371 | 22.08 | USA; Arizona; Santa Cruz County; Arivaca |
| UWBM | 105366 | 321.631 | 21.767 | USA; Louisiana; Concordia County; Three Rivers Wildlife Management Area |
| UWBM | 105367 | 353.714 | 22.44 | USA; Louisiana; Concordia County; Three Rivers Wildlife Management Area |
| UWBM | 105372 | 338.041 | 22.41 | USA; Texas; Coryell County; Jonesboro |
| UWBM | 105373 | 323.249 | 22.39 | USA; Texas; Coryell County; Jonesboro |
| UWBM | 105374 | 339.262 | 22.497 | USA; Texas; Coryell County; Jonesboro |
| UWBM | 105375 | 336.372 | 21.943 | USA; Texas; Coryell County; Jonesboro |
| UWBM | 105405 | 249.788 | 23.733 | USA; Florida; Osceola County; Three Lakes Wildlife Management Area |
| UWBM | 105412 | 330.192 | 23.067 | USA; Mississippi; Jackson County; Ward Bayou wildlife Management Area |
| UWBM | 108535 | 334.089 | 22.08 | USA; Louisiana; Caddo County; Fort Cobb Wildlife Management Area |
| UWBM | 108553 | 304.025 | 23.36 | USA; Georgia; Bryan County; Richmond Hills Wildlife Management Area |
| UWBM | 108557 | 290.097 | 21.933 | USA; Georgia; Bryan County; Richmond Hills Wildlife Management Area |
| UWBM | 108566 | 303.421 | 21.043 | USA; Georgia; Bryan County; Richmond Hills Wildlife Management Area |
| UWBM | 109275 | 344.809 | 21.323 | USA; Texas; Nacodoches County; Alazan Bayou Wildlife Management Area |
| FMNH | 110379 | 262.055 | 22.857 | USA; Illinois; Douglas County; Hinsdale |
| FMNH | 110380 | 318.549 | 24.41 | USA; Illinois; Douglas County; Hinsdale |
| UWBM | 111638 | 353.968 | 19.927 | USA; Texas; |
| UWBM | 115571 | 343.882 | 21.8 | USA; Texas; |
| UWBM | 115572 | 296.57 | 21.937 | USA; West Virginia; |
| UWBM | 115573 | 333.249 | 21.01 | USA; Texas; |
| FMNH | 163219 | 285.272 | 21.797 | USA; Georgia; Fulton County; Rosewell |
| FMNH | 163221 | 273.257 | 23.9 | USA; Kansas; Douglas County; Laurence |
| FMNH | 177251 | 288.415 | 23.657 | USA; Kansas; Douglas County; Laurence |
| FMNH | 177252 | 289.039 | 24.403 | USA; Kansas; Douglas County; Laurence |
| FMNH | 246590 | 280.58 | 23.03 | USA; Indiana; Porter County; Jackson |
| FMNH | 255339 | 236.694 | 26.177 | USA; Illinois; Kane County; Dundee |
| FMNH | 255340 | 284.607 | 22.51 | USA; Illinois; Kane County; Dundee |
| FMNH | 255341 | 295.422 | 22.78 | USA; Illinois; Cook County; Des Plaines |
| NMNH | 301714 | 322.204 | 22.49 | USA; New Jersey; Cape May |
| FMNH | 307710 | 276.137 | 23.57 | USA; New Jersey; Pisgah National Forest |
| FMNH | 307711 | 275.225 | 22.383 | USA; North Carolina; Mercer County; Princeton |
| FMNH | 327120 | 271.878 | 23.753 | USA; Illinois; Dekald County; Dekalb |
| FMNH | 327121 | 263.76 | 23.41 | USA; Illinois; Dekalb County; Dekalb |
| FMNH | 327122 | 292.674 | 24.94 | USA; Illinois; Dekalb County; Sycamore |
| FMNH | 327123 | 280.995 | 22.223 | USA; Illinois; Dekalb County; Shabbona |
| FMNH | 327124 | 294.992 | 21.127 | USA; Illinois; Dekalb County; Shabbona |
| FMNH | 327125 | 285.343 | 21.643 | USA; Illinois; Dupage County; Clarendon Hills |
| FMNH | 327126 | 273.495 | 23.41 | USA; Illinois; Dupage County; Warrenville |
| FMNH | 327127 | 297.167 | 23.123 | USA; Illinois; Dupage County; Lisle |
| FMNH | 327129 | 264.906 | 23.28 | USA; Illinios; Dupgae County; Glen Ellyn |
| FMNH | 327130 | 276.875 | 21.397 | USA; Illinois; Dupage County; Villa Park |
| FMNH | 327134 | 295.871 | 23.483 | USA; Illinois; Dupage County; Glenn Ellyn |
| FMNH | 327137 | 309.233 | 21.553 | USA; Illinois; Dupage County; Glenn Ellyn |
| FMNH | 327138 | 297.928 | 24.143 | USA; Illinois; Dupage County; Glenn Ellyn |
| FMNH | 327140 | 282.755 | 21.6 | USA; Illinois; Dupage County; Glenn Ellyn |
| FMNH | 327141 | 297.092 | 20.913 | USA; Illinois; Dupage County; Wheaton |
| FMNH | 327142 | 291.294 | 25.617 | USA; Illinois; Dupage County; Wheaton |
| FMNH | 327143 | 250.43 | 22.637 | USA; Illinois; Dupage County |
| FMNH | 327145 | 288.75 | 23.693 | USA; Illinois; Ogle County; Byren |
| FMNH | 327146 | 302.277 | 24.26 | USA; Illinois; Alexander County; Olive Branch |
| FMNH | 327147 | 294.975 | 23.917 | USA; Illinois; Alexander County; Olive Branch |
| FMNH | 327148 | 269.158 | 24.007 | USA; Illinios; Alexander County; Thebes |
| FMNH | 327149 | 289.383 | 13.167 | USA; Illinois; McHenry County; Woodstock |
| FMNH | 327150 | 292.458 | 23.44 | USA; Illinois; McHenry County; Crystal Lake |
| FMNH | 327153 | 309.11 | 23.98 | USA; Illinois; Cook County; Hickory Hills |
| FMNH | 334737 | 280.835 | 22.477 | USA; Illinois; Cook County; Chicago |
| NMNH | 337660 | 294.157 | 21.32 | USA; Kentucky; Pike County; Belfry |
| NMNH | 337662 | 316.986 | 21.4 | USA; Kentucky; Wayne County; Monticello |
| NMNH | 337663 | 286.253 | 20.97 | USA; Georgia; Fulton County; Hickman |
| NMNH | 337664 | 297.674 | 22.243 | USA; Georgia; Fulton County; Hickman |
| NMNH | 337665 | 335.85 | 21.513 | USA; Kentucky; Wayne County; Rocky Branch |
| NMNH | 337667 | 302.056 | 22.18 | USA; Kentucky; Lewis County; Quincy |
| NMNH | 337837 | 280.626 | 21.72 | USA; Kentucky; Meade County; Brandenburg |
| NMNH | 337838 | 302.956 | 23.727 | USA; Kentucky; Union County; Waverly |
| NMNH | 337839 | 286.791 | 21.69 | USA; Kentucky; Meade County; Rock Haven |
| NMNH | 337840 | 300.079 | 22.767 | USA; Kentucky; Union County; Union Town |
| NMNH | 338040 | 284.397 | 21.947 | USA; Georgia; Seminole County; Chattahoochee |
| NMNH | 338042 | 273.599 | 20.16 | USA; Georgia; Seminole County; Chattahoochee |
| NMNH | 338043 | 277.333 | 20.717 | USA; Georgia; Towns County; Young Harris |
| NMNH | 338044 | 296.268 | 22.617 | USA; Georgia; Seminole County; Chattahoochee |
| NMNH | 338046 | 270.116 | 23.167 | USA; Georgia; Earl County; Blakely |
| NMNH | 338047 | 267.692 | 22.04 | USA; Georgia; Earl County; Blakely |
| NMNH | 338048 | 307.538 | 21.733 | USA; North Carolina; Carteret County; Beaufort |
| MCZ | 338830 | 277.477 | 23.15 | USA; Texas; Dickens County; Afton |
| MCZ | 338831 | 304.252 | 21.407 | USA; Texas; Dickens County; Afton |
| MCZ | 338832 | 273.765 | 23.373 | USA; Texas; Dickens County; Afton |
| MCZ | 338833 | 240.721 | 21.53 | USA; Texas; Dickens County; Afton |
| NMNH | 339070 | 319.838 | 20.897 | USA; South Carolina; Georgetown County; Murrells Inlet |
| NMNH | 339334 | 278.8 | 22.287 | USA; Georgia; Clarke County; Athens |
| NMNH | 339336 | 306.266 | 22.113 | USA; Georgia; Earl County; Blakely |
| NMNH | 339337 | 306.376 | 23.493 | USA; Georgia; Earl County; Blakely |
| NMNH | 339338 | 292.723 | 23.653 | USA; Georgia; Earl County; Blakely |
| NMNH | 339340 | 267.711 | 22.123 | USA; Georgia; Towns County; Young Harris |
| NMNH | 339342 | 304.313 | 22.697 | USA; Alabama; Lawrence County; Moulton |
| NMNH | 339345 | 269.411 | 20.42 | USA; South Carolina; Greenwood County; Greenwood |
| NMNH | 339346 | 297.391 | 22.733 | USA; South Carolina; Dorschester County; Harleyville |
| NMNH | 339347 | 244.805 | 23.107 | USA; South Carolina; Abbeville County |
| NMNH | 339348 | 260.243 | 21.193 | USA; Georgia; Chatham County; Savannah |
| NMNH | 339350 | 287.429 | 19.67 | USA; Virginia; Princess Anne County; Pungo |
| NMNH | 339351 | 329.74 | 22.01 | USA; North Carolina; Durham County; Durham |
| NMNH | 339352 | 280.54 | 21.233 | USA; Georgia; Burke County; Waynesboro |
| NMNH | 339353 | 265.52 | 22.567 | USA; South Carolina; Charleston County; Mount Pleasant |
| NMNH | 339860 | 315.512 | 21.283 | USA; South Carolina; Greenville County; Saluda Gap |
| NMNH | 339861 | 315.199 | 21.67 | USA; Kentucky; Jefferson County; Louisville |
| NMNH | 339862 | 291.657 | 22.313 | USA; Virginia; Fairfax County; Alexandria |
| NMNH | 339863 | 296.635 | 20.903 | USA; Virginia; Fairfax County; Alexandria |
| NMNH | 339864 | 275.073 | 19.963 | USA; North Carolina; Buncombe County; Asheville |
| NMNH | 340208 | 294.937 | 21.277 | USA; Virginia; Accomack County; Chincoteague |
| NMNH | 341433 | 314.25 | 22.383 | USA; Virginia; Fairfax County; Belvoir |
| NMNH | 341474 | 256.692 | 19.74 | USA; Pennsylvannia; Allengheny County; Pittsburgh |
| FMNH | 341669 | 278.43 | 21.897 | USA; Illinois; Cook County; Chicago |
| NMNH | 341847 | 272.096 | 21.69 | USA; South Carolina; Greenville County; Saluda Gap |
| NMNH | 341848 | 339.983 | 23.097 | USA; Tennessee; Hamblen County; Morristown |
| NMNH | 341849 | 287.043 | 20.67 | USA; North Carolina; Carteret County; Beaufort |
| NMNH | 341850 | 326.649 | 23.093 | USA; Delaware; New Castle County; Glasgow |
| NMNH | 341853 | 309.108 | 22.55 | USA; Maryland; District of Columbia; Washington D.C. |
| NMNH | 341854 | 313.17 | 22.77 | USA; Alabama; Houston County; Dothan |
| NMNH | 348747 | 266.763 | 20.817 | USA; West Virginia; Calhoun County; Rocksdale |
| NMNH | 348748 | 278.864 | 20.577 | USA; West Virginia; Calhoun County; Arnoldsburg |
| NMNH | 348749 | 291.774 | 21.327 | USA; West Virginia; Nicholas County; Gilboa |
| NMNH | 348750 | 280.088 | 20.093 | USA; West Virginia; Calhoun County; Big Springs |
| NMNH | 348751 | 287.621 | 21.657 | USA; West Virginia; Logan County |
| NMNH | 348753 | 297.722 | 21.247 | USA; West Virginia; Wayne County; Dunlow |
| NMNH | 348754 | 291.501 | 21.55 | USA; West Virginia; Cabell County; Huntington |
| NMNH | 348755 | 275.543 | 19.97 | USA; West Virginia; Huntington County |
| NMNH | 348756 | 274.547 | 22.74 | USA; West Virginia; Barbour County; Phillippi |
| NMNH | 348757 | 313.462 | 22.357 | USA; West Virginia; Barbour County; Phillippi |
| NMNH | 349351 | 279.359 | 18.41 | USA; West Virginia; Pocahontas County; Williams River |
| NMNH | 349354 | 281.811 | 21.737 | USA; West Virginia; Cabell County; Barboursville |
| NMNH | 349356 | 257.28 | 21.69 | USA; West Virginia; Mason County |
| NMNH | 349357 | 286.452 | 21.537 | USA; West Virginia; Mason County; Mercer's Bottom |
| FMNH | 351316 | 284.609 | 22.687 | USA; Wisconsin; Brown County; Little Suamico |
| FMNH | 351317 | 271.209 | 22.53 | USA; Wisconsin; Brown County; Bay Beach Wildlife Sanctuary |
| FMNH | 351318 | 309.617 | 22.66 | USA; Wisconsin; Brown County; De Pere |
| NMNH | 351473 | 285.782 | 21.41 | USA; Tennessee; Shelby County; Frayser |
| NMNH | 351474 | 280.48 | 19.273 | USA; Tennessee; Obion County; Reelfoot Lake |
| NMNH | 351476 | 323.45 | 21.34 | USA; Tennessee; Wayne County; Waynesboro |
| NMNH | 351477 | 268.89 | 21.333 | USA; Tennessee; Johnson County; Shady Valley |
| NMNH | 351478 | 314.969 | 22.377 | USA; Tennessee; Cocke County; Cosby |
| NMNH | 351479 | 265.924 | 20.432 | USA; Tennessee; Fayette County |
| NMNH | 351480 | 308.375 | 21.54 | USA; Tennessee; Obion County; Reelfoot Lake |
| NMNH | 351481 | 302.344 | 22.38 | USA; Tennessee; Johnson County; Shady Valley |
| FMNH | 352310 | 243.798 | 22.477 | USA; New Jersey; Burlington County; Medford |
| FMNH | 352311 | 286.945 | 23.45 | USA; Virginia; Bototourt County; Roanoke |
| FMNH | 352312 | 249.902 | 21.353 | USA; Kentucky; McCracken County; Paducah |
| FMNH | 352313 | 261.092 | 23.287 | USA; Kentucky; McCracken County; Paducah |
| FMNH | 352314 | 317.189 | 24.21 | USA; Kentucky; McCracken County; Paducah |
| NMNH | 352470 | 302.894 | 22.363 | USA; Tennessee; Lake County; Phillipy |
| NMNH | 352474 | 264.109 | 21.13 | USA; Tennessee; Lincoln County; Frankewing |
| NMNH | 352475 | 337.712 | 22.5 | USA; Tennessee; Stewart County; Dover |
| NMNH | 353168 | 303.192 | 21.287 | USA; Kentucky; Bell County; Middlesboro |
| NMNH | 353169 | 251.556 | 21.987 | USA; Kentucky; Bell County; Middlesboro |
| NMNH | 353171 | 284.279 | 23.26 | USA; Kentucky; Boone County; Burlington |
| NMNH | 353172 | 283.246 | 21.73 | USA; Kentucky; Butler County; Round Hill |
| NMNH | 353175 | 307.581 | 21.707 | USA; Kentucky; Trigg County; Golden Pond |
| NMNH | 353178 | 310.779 | 21.363 | USA; Kentucky; Trigg County; Canton |
| FMNH | 354856 | 246.893 | 22.177 | USA; Illinois; Cook County; Chicago |
| FMNH | 356952 | 301.534 | 23.297 | USA; Minnesota; Ramsey County; St. Paul |
| FMNH | 357126 | 271.631 | 21.513 | USA; Minnesota; Washington County; Aftron |
| NMNH | 357899 | 320.257 | 21.833 | USA; North Carolina; Ashe County; West Jefferson |
| NMNH | 357900 | 272.707 | 22.47 | USA; North Carolina; Macon County; Franklin |
| NMNH | 357901 | 283.319 | 20.907 | USA; North Carolina; Cherokee County; Murphy |
| NMNH | 357903 | 264.221 | 21.153 | USA; North Carolina; Pasquotank County; South mills |
| NMNH | 357904 | 295.879 | 23.09 | USA; North Carolina; Pasquotank County; South mills |
| NMNH | 357905 | 284.281 | 22.237 | USA; North Carolina; Sampson County; Clinton |
| NMNH | 357906 | 292.645 | 22.62 | USA; North Carolina; Sampson County; Clinton |
| NMNH | 357907 | 265.535 | 20.797 | USA; North Carolina; Brunswick County; Southport |
| NMNH | 357908 | 297.518 | 21.133 | USA; North Carolina; Brunswick County; Southport |
| NMNH | 357909 | 315.651 | 22.64 | USA; North Carolina; Brunswick County; Smith Island |
| NMNH | 357910 | 319.497 | 22.963 | USA; North Carolina; Brunswick County; Smith Island |
| NMNH | 357911 | 264.645 | 22.217 | USA; North Carolina; Brunswick County; Smith Island |
| NMNH | 357913 | 308.965 | 20.987 | USA; North Carolina; Anson County; Wadesboro |
| NMNH | 358487 | 308.225 | 24.163 | USA; North Carolina; Iredell County; Statesville |
| NMNH | 358489 | 363.382 | 22.89 | USA; North Carolina; Rockingham County; Reidsville |
| NMNH | 358491 | 275.373 | 20.1 | USA; North Carolina; Catawba County; Catawba |
| NMNH | 362723 | 302.427 | 22.78 | USA; South Carolina; Newberry County; Whitmire |
| NMNH | 362724 | 311.719 | 21.87 | USA; South Carolina; Newberry County; Whitmire |
| NMNH | 362725 | 279.696 | 22.2 | USA; South Carolina; Pickens County; Marietta |
| NMNH | 362726 | 305.884 | 22.307 | USA; South Carolina; Oconee County; Walhalla |
| NMNH | 362727 | 255.87 | 21.333 | USA; South Carolina; Oconee County; Walhalla |
| NMNH | 362728 | 262.925 | 21.843 | USA; South Carolina; Edgefield County; Edgefield |
| NMNH | 362729 | 324.124 | 22.283 | USA; South Carolina; Beaufort County; Bluffton |
| NMNH | 362730 | 282.468 | 22.343 | USA; South Carolina; Beaufort County; Bluffton |
| NMNH | 362732 | 261.769 | 21.693 | USA; South Carolina; Dorchester County; St. George |
| NMNH | 362733 | 269.812 | 20.95 | USA; South Carolina; Dorchester County; St. George |
| NMNH | 362734 | 332.037 | 22.767 | USA; South Carolina; McCormick County |
| NMNH | 362735 | 282.609 | 22.663 | USA; South Carolina; McCormick County; McCormick |
| NMNH | 362739 | 296.196 | 21.227 | USA; South Carolina; Chester County; Chester |
| NMNH | 362742 | 304.795 | 22.353 | USA; South Carolina; Marloboro County; Bennettsville |
| NMNH | 362743 | 288.347 | 22.427 | USA; South Carolina; Allendale County; Allendale |
| NMNH | 362744 | 266.128 | 21.223 | USA; South Carolina; Allendale County; Allendale |
| NMNH | 362746 | 322.961 | 21.85 | USA; South Carolina; Lee County; Lynchburg |
| NMNH | 362747 | 305.366 | 20.393 | USA; South Carolina; Charleston County; McClellanville |
| NMNH | 363164 | 288.605 | 24.047 | USA; Mississippi; Lowndes County; Columbus |
| NMNH | 363167 | 289.036 | 24.483 | USA; Mississippi; Harrison County; Cat Island |
| NMNH | 363169 | 301.901 | 22.667 | USA; North Carolina; Durham County; Durham |
| NMNH | 363170 | 265.573 | 20.857 | USA; North Carolina; Durham County; Durham |
| NMNH | 363171 | 295.587 | 22.557 | USA; North Carolina; McDowell County; Marion |
| NMNH | 363172 | 310.375 | 21.043 | USA; Kentucky; Warren County; Bowling Green |
| NMNH | 363173 | 302.696 | 22.352 | USA; Pennsylvannia; Allengheny County; Pittsburgh |
| NMNH | 363174 | 269.251 | 20.787 | USA; South Carolina; Charleston County; Mount Pleasant |
| NMNH | 363176 | 306.984 | 21.067 | USA; Georgia; Earl County; Blakely |
| NMNH | 363178 | 311.064 | 22.66 | USA; Georgia; Towns County; Young Harris |
| NMNH | 363180 | 296.895 | 21.957 | USA; Georgia; Chatham County; Savannah |
| FMNH | 363683 | 268.847 | 21.6 | USA; Wisconsin; Brown County; Green Bay |
| FMNH | 363684 | 296.281 | 22.903 | USA; Wisconsin; Brown County; Green Bay |
| FMNH | 363685 | 291.058 | 22.89 | USA; Wisconsin; Brown County; Green Bay |
| FMNH | 363686 | 219.297 | 19.417 | USA; Wisconsin; Brown County; Green Bay |
| FMNH | 364096 | 268.168 | 21.503 | USA; Illinois; Will County; Bolingbrook |
| MCZ | 364211 | 251.955 | 21.827 | USA; Texas; Dickens County; Afton |
| MCZ | 364212 | 247.494 | 21.587 | USA; Texas; Dickens County; Afton |
| MCZ | 364213 | 211.365 | 22.97 | USA; Texas; Dickens County; Afton |
| MCZ | 364214 | 276.939 | 22.157 | USA; Texas; Dickens County; Afton |
| NMNH | 367556 | 260.901 | 21.893 | USA; Delaware; New Castle County; Glasgow |
| NMNH | 367557 | 267.951 | 23.727 | USA; Mississippi; Hinds County; Jackson |
| NMNH | 367559 | 280.172 | 21.407 | USA; Mississippi; Bolivar County; Rosedale |
| NMNH | 367560 | 292.846 | 22.04 | USA; Alabama; Mobile County; Mobile |
| NMNH | 367561 | 301.409 | 21.547 | USA; Florida; Escambia County; Pensacola |
| NMNH | 367562 | 270.842 | 23.36 | USA; Florida; Monroe County; Key Largo |
| NMNH | 367563 | 309.474 | 22.607 | USA; Louisiana; New Orleans County; New Orleans |
| NMNH | 367781 | 275.133 | 20.477 | USA; Georgia; Clarke County; Athens |
| NMNH | 367791 | 279.744 | 20.72 | USA; North Carolina; Buncombe County; Asheville |
| NMNH | 367967 | 263.92 | 21.39 | USA; Ohio; Clermont County; Union |
| NMNH | 371227 | 278.522 | 19.557 | USA; Georgia; Grady County; Sherwood Plantation |
| FMNH | 374306 | 330.263 | 24.483 | USA; Pennsylvannia; Butler County; Butler |
| FMNH | 374307 | 303.603 | 21.983 | USA; Pennsylvannia; Westmoreland County; North Huntington |
| NMNH | 378843 | 292.283 | 20.737 | USA; Maryland; Prince Georges County; Clinton |
| NMNH | 379300 | 269.825 | 21.197 | USA; Georgia; Earl County; Blakely |
| NMNH | 380005 | 245 | 21.403 | USA; Maryland; District of Columbia; Washington D.C. |
| NMNH | 380431 | 301.875 | 21.653 | USA; Mississippi; Harrison County; Saucier |
| NMNH | 380432 | 253.32 | 20.127 | USA; Mississippi; Harrison County; Deer Island |
| NMNH | 380433 | 266.988 | 21.613 | USA; Georgia; Clarke County; Winterville |
| NMNH | 380434 | 301.872 | 22.547 | USA; Georgia; Morgan County; Madison |
| NMNH | 380435 | 255.648 | 21.36 | USA; Georgia; Barrow County; Bethlehem |
| NMNH | 380436 | 276.987 | 22.367 | USA; Georgia; Charlton County; Folkston |
| NMNH | 380437 | 285.686 | 20.773 | USA; Georgia; Charlton County; Folkston |
| NMNH | 380438 | 295.634 | 21.507 | USA; Georgia; Clarke County; Athens |
| NMNH | 380439 | 302.415 | 21.037 | USA; Georgia; Laurens County; Dublin |
| NMNH | 382285 | 309.327 | 22.073 | USA; Georgia; Madison County; Pocatalago |
| NMNH | 382286 | 289.125 | 23.843 | USA; Georgia; Dekalb County; Lithonia |
| NMNH | 382289 | 276.56 | 23.26 | USA; Georgia; Liberty County; Yellow Bluff |
| NMNH | 382290 | 307.72 | 21.67 | USA; Georgia; Liberty County; Yellow Bluff |
| NMNH | 382291 | 289.075 | 22.57 | USA; Georgia; Liberty County; Yellow Bluff |
| NMNH | 382292 | 240.241 | 20.803 | USA; Georgia; Liberty County; Midway |
| NMNH | 382293 | 285.792 | 22.15 | USA; Georgia; Liberty County; Hinesville |
| NMNH | 382294 | 260.117 | 20.693 | USA; Georgia; Montgomery County; Tarrytown |
| NMNH | 382296 | 272.755 | 22.74 | USA; Georgia; Wheeler County; Alamo |
| NMNH | 382465 | 285.405 | 20.693 | USA; Georgia; Long County; Hinesville |
| NMNH | 382929 | 284.929 | 22.94 | USA; Georgia; Dekalb County; Decatur |
| NMNH | 382930 | 292.556 | 20.397 | USA; Georgia; Dekalb County; Decatur |
| FMNH | 385942 | 332.376 | 24.337 | USA; Illinois; Dupage County; Elmhurst |
| FMNH | 386024 | 269.274 | 22.633 | USA; Illinois; Cook County; Palatine |
| FMNH | 386027 | 303.239 | 19.09 | USA; Illinois; Cook County; Palatine |
| FMNH | 387865 | 270.465 | 21.883 | USA; Wisconsin; Brown County; Green Bay |
| NMNH | 393682 | 311.473 | 22.333 | USA; Georgia; Whitefield County; Dalton |
| NMNH | 394912 | 286.786 | 21.997 | USA; Georgia; Marion County; Buena Vista |
| NMNH | 394913 | 303.165 | 22.3 | USA; Tennessee; Shelby County; Memphis |
| NMNH | 394914 | 301.902 | 21.29 | USA; Oklahoma; Tulsa County; Tulsa |
| FMNH | 395370 | 291.959 | 23.167 | USA; Wisconsin; Racine County; Burlington |
| FMNH | 395371 | 277.331 | 22.217 | USA; Wisconsin; Racine County; Burlington |
| FMNH | 395372 | 287.939 | 23.247 | USA; Illinois; Cook County; Westchester |
| FMNH | 397087 | 235.722 | 21.657 | USA; Illinois; Dupage County |
| NMNH | 421989 | 310.623 | 21.623 | USA; Maryland; Talbot County; Trappe |
| NMNH | 422143 | 289.234 | 23.543 | USA; Maryland; Dorchester County; Salem |
| NMNH | 422198 | 310.025 | 22.09 | USA; Delaware; Kent County; Milford |
| NMNH | 422404 | 283.236 | 22.427 | USA; Delware; Sussex County; Millsboro |
| NMNH | 422868 | 299.3 | 21.51 | USA; Maryland; Prince George County |
| FMNH | 430218 | 267.269 | 23.337 | USA; Illinois; Cook County; Forest Park |
| FMNH | 430272 | 244.678 | 21.193 | USA; Wisconsin; Brown County; Bay Beah Wildlife Sanctuary |
| FMNH | 430298 | 285.891 | 22.44 | USA; Minnesota; Fillmore County; Preston |
| NMNH | 433555 | 292.474 | 21.873 | USA; Virginia; Russell County; Lebanon |
| FMNH | 435014 | 297.428 | 22.4 | USA; Illinois; Dupage County; Naperville |
| FMNH | 435161 | 265.698 | 24.31 | USA; Illinois; Cook County; Chicago |
| FMNH | 435162 | 265.395 | 22.133 | USA; Illinios; Cook County; Chicago |
| NMNH | 437599 | 295.02 | 21.11 | USA; Virginia; Montgomery County; Blacksburg |
| FMNH | 439769 | 281.201 | 24.487 | USA; Illinois; Cook County; Westchester |
| FMNH | 440101 | 268.31 | 23.227 | USA; Minnesota; Winona County |
| FMNH | 440245 | 259.958 | 24.057 | USA; Illinois; Kendall; Oswego |
| FMNH | 440246 | 282.635 | 22.15 | USA; Illinois; Dupage Conty; Glen Ellyn |
| FMNH | 448962 | 262.729 | 21.933 | USA; Illinois; Cook County; Chicago |
| FMNH | 452395 | 299.127 | 22.847 | USA; Illinois; Cook County; Chicago |
| FMNH | 454294 | 306.123 | 23.513 | USA; Illinois; Dupage County; Naperville |
| FMNH | 454295 | 259.168 | 23.447 | USA; Illinois; Cook County; Oak Park |
| FMNH | 454296 | 238.969 | 23.127 | USA; Illinois; Dupage County; Glenn Ellyn |
| FMNH | 456370 | 299.503 | 23.42 | USA; Illinois; Cook County; Chicago |
| FMNH | 456404 | 244.32 | 20.677 | USA; Illinois; Cook County; Skokie |
| FMNH | 458985 | 245.757 | 23.673 | USA; Illinois; Cook County; Chicago |
| FMNH | 458987 | 266.153 | 22.39 | USA; Illinois; Cook County; Northbrook |
| FMNH | 459047 | 320.756 | 25.01 | USA; Minnesota; Crow Wing County; Nisswa |
| FMNH | 461292 | 265.726 | 24.62 | USA; Illinois; Dupage County; Bloomigdale |
| NMNH | 461702 | 283.984 | 21.417 | USA; Maryland; Harford County; Army Chemical Center |
| FMNH | 461766 | 280.395 | 22.4 | USA; Wisconsin; Brown County; Bay Beach Wildlife Sanctuary |
| NMNH | 462617 | 267.172 | 19.933 | USA; Maryland; District of Columbia; Washington D.C. |
| FMNH | 463266 | 286.529 | 23.113 | USA; Illinois; Dupage County; Elmhurst |
| FMNH | 463267 | 258.145 | 23.443 | USA; Illinois; Cook County; Oak Park |
| FMNH | 464375 | 282.585 | 23.44 | USA; Illinois; Cook County; Chicago |
| FMNH | 464376 | 266.28 | 23.12 | USA; Illinios; Dupage County; Darien |
| NMNH | 466419 | 279.255 | 21.223 | USA; Virginia; Fairfax County; Alexandria |
| NMNH | 466420 | 280.045 | 21.003 | USA; Maryland; Prince George County; Bladensburg |
| NMNH | 466569 | 319.839 | 23.213 | USA; Oklahoma; Johnston County; Tishomingo |
| NMNH | 466574 | 263.597 | 21.55 | USA; Virginia; Fairfax County; Alexandria |
| FMNH | 466629 | 273.797 | 22.42 | USA; Illinois; Cook County; South Barrington |
| FMNH | 466907 | 294.458 | 24.843 | USA; Illinois; Cook County |
| FMNH | 466909 | 296.871 | 20.113 | USA; Illinois; Cook County; Palos Park |
| FMNH | 466910 | 273.871 | 22.64 | USA; Illinois; Kane County; Batavia |
| FMNH | 467812 | 301.291 | 22.04 | USA; Illinois; Dupage County; Davien |
| FMNH | 470046 | 250.399 | 24.48 | USA; Illinois; Cook County; Chicago |
| FMNH | 470047 | 253.014 | 24.483 | USA; Illinois; Cook County; Chicago |
| FMNH | 470444 | 245.376 | 21.693 | USA; Wisconsin; Brown County |
| NMNH | 470756 | 267.134 | 21.363 | USA; New Jersey; Atlantic County; Oceanville |
| FMNH | 473029 | 288.657 | 22.777 | USA; Ohio; Cuyahoga County; North Royalton |
| FMNH | 473030 | 273.561 | 23.413 | USA; Illinois; Cook County; Chicago |
| FMNH | 473031 | 289.783 | 22.207 | USA; Illinois; Cook County; Skokie |
| FMNH | 473032 | 273.04 | 21.76 | USA; Illinois; Cook County; Chicago |
| FMNH | 477489 | 317.662 | 24.633 | USA; Illinois; Dupage County; Glen Ellyn |
| FMNH | 477490 | 248.735 | 22.75 | USA; Wisconsin; Brown County; Bay Beach Wildlife Sanctuary |
| NMNH | 477901 | 306.062 | 22.93 | USA; Virginia; Fairfax County; Annandale |
| NMNH | 477903 | 269.068 | 20.873 | USA; Maryland; District of Columbia; Washington D.C. |
| NMNH | 477904 | 279.639 | 20.56 | USA; North Carolina; Buncombe County; Asheville |
| NMNH | 478727 | 297.702 | 22.113 | USA; Florida; Leon County |
| NMNH | 478729 | 326.529 | 21.023 | USA; Florida; Leon County |
| NMNH | 478730 | 283.28 | 22.253 | USA; Florida; Leon County |
| NMNH | 478732 | 276.646 | 22.637 | USA; Florida; Leon County |
| NMNH | 479150 | 321.793 | 21.35 | USA; Conneticut; New Haven County; New Haven |
| NMNH | 480458 | 296.418 | 22.093 | USA; New Mexico; Sheridan County; Niobrara |
| NMNH | 480725 | 295.692 | 21.92 | USA; Michigan; Washtenaw County; Ann Arbor |
| NMNH | 481350 | 267.677 | 21.52 | USA; Maryland; District of Columbia; Washington D.C. |
| FMNH | 488480 | 312.137 | 23.81 | USA; Illinois; Kane County; St. Charles |
| FMNH | 491315 | 260.287 | 22.343 | USA; Illinois; Dupage County; Naperville |
| FMNH | 491316 | 270.619 | 22.883 | USA; Illinois; Dupage County; Naperville |
| FMNH | 492390 | 298.016 | 24.317 | USA; Wisconsin; Brown County; Bay Beach Wildlife Sanctuary |
| FMNH | 495702 | 267.553 | 23.44 | USA; Minnesota; Crow Wing County; Brainerd |
| FMNH | 496650 | 273.579 | 24.38 | USA; Wisconsin; Walworth County; Lake Geneva |
| FMNH | 498425 | 247.262 | 22.557 | USA; Illinois; Dupage County; Oak Brook |
| NMNH | 525265 | 297.705 | 22.053 | USA; Maryland; District of Columbia; Washington D.C. |
| NMNH | 526146 | 280.91 | 21.8 | USA; Virginia; Arlington County; Ballston |
| NMNH | 529342 | 261.87 | 21.567 | USA; South Carolina; Georgetown County; Georgetown |
| NMNH | 529343 | 302.248 | 21.747 | USA; South Carolina; Georgetown County; Georgetown |
| NMNH | 529344 | 267.259 | 20.79 | USA; Michigan; Monroe County; Erie |
| NMNH | 529612 | 315.739 | 21.72 | USA; Virginia; Loudoun County; Ashburn |
| NMNH | 529619 | 285.79 | 21.733 | USA; Virginia; Fairfax County; S. Falls Church |
| NMNH | 529663 | 271.08 | 21.57 | USA; Maryland; Montgomery County; Glen Echo |
| NMNH | 529676 | 288.072 | 24.153 | USA; Maryland; Montgomery County; Gaithersburg |
| NMNH | 529686 | 252.592 | 22.473 | USA; Maryland; District of Columbia; Washington D.C. |
| NMNH | 532199 | 286.318 | 21.417 | USA; Maryland; Montgomery County; Gaithersburg |
| NMNH | 532861 | 277.338 | 22.317 | USA; Maryland; Montgomery County; Chevy Chase |
| NMNH | 564178 | 288.747 | 20.757 | USA; Indiana; Comanche County; Wichita Wildlife Research Station |
| NMNH | 564179 | 267.533 | 21.427 | USA; Indiana; Clay County; Brazil |
| NMNH | 564385 | 274.85 | 21.17 | USA; Virginia; Fairfax County; Lorton |
| NMNH | 564386 | 279.106 | 23.433 | USA; Virginia; Fairfax County; Lorton |
| NMNH | 564387 | 276.676 | 20.833 | USA; Virginia; Fairfax County; Lorton |
| NMNH | 564440 | 302.991 | 21.187 | USA; Virginia; Fairfax County; Lorton |
| NMNH | 564845 | 286.365 | 20.773 | USA; Virginia; Fairfax County; Falls Church |
| NMNH | 565235 | 257.841 | 22.777 | USA; Virginia; Fairfax County; Alexandira |
| NMNH | 565963 | 295.369 | 21.897 | USA; Virginia; Prince William County; Manassas |
| NMNH | 566159 | 296.67 | 22.94 | USA; Michigan; Ingham County; Lansing |
| NMNH | 566650 | 293.601 | 21.553 | USA; Virginia; Fairfax County; Alexandria |
| NMNH | 566982 | 276.771 | 21.807 | USA; Maryland; Montgomery County; Potomac |
| NMNH | 566984 | 270.635 | 20.79 | USA; Maryland; Montgomery County; Potomac |
| NMNH | 567101 | 315.773 | 21.71 | USA; Virginia; Fairfax County; Great Falls |
| NMNH | 567226 | 295.197 | 21.19 | USA; West Virginia; Hampshire County; Romney |
| NMNH | 567361 | 322.885 | 21.367 | USA; New Jersey; Burlington County; Medford |
| NMNH | 567486 | 258.126 | 21.19 | USA; Illinois; Peoria County; Mossville |
| NMNH | 567488 | 266.007 | 22.273 | USA; Illinios; Peoria County; Chillicothe |
| NMNH | 567489 | 278.184 | 21.447 | USA; Illinios; Peoria County; Chillicothe |
| NMNH | 567642 | 274.754 | 20.237 | USA; Illinois; Peoria County; Chillicothe |
| NMNH | 567865 | 274.078 | 20.92 | USA; Indiana; Tippecanoe County; West Lafayette |
| NMNH | 571159 | 301.928 | 22.217 | USA; Virginia; Arlington County; Potomac |
| NMNH | 573019 | 284.1 | 22.81 | USA; Maryland; Howard County; Columbia |
| NMNH | 573561 | 273.627 | 20.737 | USA; Maryland; Montgomery County; Potomac |
| NMNH | 576932 | 301.844 | 22.313 | USA; New York; Onondaga County; Dewitt |
| NMNH | 577134 | 237.462 | 22.553 | USA; Ohio; Jefferson County; Steubenville |
| NMNH | 582703 | 260.965 | 21.57 | USA; Maryland; Talbot County; Bozman |
| NMNH | 582704 | 283.576 | 21.657 | USA; Maryland; Talbot County; Bozman |
| NMNH | 582705 | 277.262 | 23.6 | USA; Maryland; Talbot; Easton |
| NMNH | 582706 | 286.684 | 22.113 | USA; Maryland; Talbot County; St. Michaels |
| NMNH | 582707 | 285.061 | 23.283 | USA; Maryland; Talbot County; Royal Oak |
| NMNH | 582708 | 264.853 | 21.35 | USA; Maryland; Talbot County; Royal Oak |
| NMNH | 582709 | 276.331 | 22.537 | USA; Maryland; Talbot County; Royal Oak |
| NMNH | 582710 | 274.06 | 23.04 | USA; Maryland; Talbot County; Bozman |
| NMNH | 582711 | 271.222 | 23.723 | USA; Maryland; Queen Annes County; Church Hill |
| NMNH | 582712 | 277.282 | 21.367 | USA; Maryland; Queen Annes County; Grasonville |
| NMNH | 582884 | 255.739 | 21.943 | USA; Maryand; Frederick County; Little Falls |
| NMNH | 593503 | 346.704 | 22.81 | USA; Arizona; Nevada County; Prescott |
| NMNH | 593505 | 308.772 | 22.473 | USA; Arizona; Nevada County; Prescott |
| NMNH | 593506 | 320.989 | 22.34 | USA; Arizona; Monroe County; Brinkley |
| NMNH | 593518 | 318.19 | 21.89 | USA; Maryland; Prince Georges County; Beltsville |
| NMNH | 593533 | 299.74 | 22.55 | USA; Texas; Stonewall County; Aspenmont |
| NMNH | 593534 | 318.034 | 21.117 | USA; Texas; Lee County; Lincoln |
| NMNH | 593535 | 285.625 | 22.053 | USA; Virginia; Stafford County; Stafford |
| NMNH | 593536 | 297.402 | 21.14 | USA; Virginia; Stafford County; Stafford |
| NMNH | 593537 | 285.855 | 20.797 | USA; Virginia; Fairfax County; Oakton |
| NMNH | 593538 | 266.568 | 20.493 | USA; Virginia; Fairfax County; Oakton |
| NMNH | 593539 | 257.943 | 20.63 | USA; Virginia; Fairfax County; Oakton |
| NMNH | 593540 | 275.067 | 21.787 | USA; Virginia; Fairfax County; Oakton |
| NMNH | 593541 | 240.852 | 20.353 | USA; Virginia; Fairfax County; Alexandria |
| NMNH | 593542 | 278.593 | 23.023 | USA; Virginia; Fairfax County; Oakton |
| NMNH | 596181 | 277.248 | 21.88 | USA; Virginia; Rappahannock County; Flint Hill |
| NMNH | 596285 | 289.301 | 21.82 | USA; Maryland; Montgomery County; Potomac |
| NMNH | 596365 | 283.645 | 19.713 | USA; Maryland; Charles County; Waldorf |
| NMNH | 596541 | 270.726 | 21.63 | USA; Maryland; Montgomery County; Chevy Chase |
| NMNH | 596542 | 288.758 | 22.77 | USA; Maryland; Montgomery County; Potomac |
| NMNH | 596908 | 284.56 | 24.32 | USA; Virginia; Lousia County |
| NMNH | 597115 | 286.334 | 21.913 | USA; Virginia; Prince William County; Manassas |
| NMNH | 597323 | 288.006 | 21.297 | USA; Virginia; Fairfax County |
| NMNH | 597335 | 306.498 | 22.363 | USA; West Virginia; Jefferson County; Halltown |
| NMNH | 597635 | 264.402 | 22.473 | USA; Virginia; Fairfax County; Annandale |
| NMNH | 597801 | 290.997 | 22.467 | USA; Maryland; St. Marys County; Leonardtown |
| NMNH | 598283 | 312.616 | 22.783 | USA; Maryland; Talbot County; Easton |
| NMNH | 598284 | 295.132 | 22.953 | USA; Maryland; Talbot County; Easton |
| NMNH | 598285 | 270.234 | 23.22 | USA; Maryland; Talbot County; Easton |
| NMNH | 598286 | 255.34 | 21.507 | USA; Maryland; Talbot County; Easton |
| NMNH | 598287 | 287.384 | 22.087 | USA; Maryland; Talbot County; Easton |
| NMNH | 598288 | 269.961 | 22.157 | USA; Maryland; Talbot County; Easton |
| NMNH | 598289 | 271.001 | 21.16 | USA; Maryland; Talbot County; Easton |
| NMNH | 598290 | 290.936 | 21.533 | USA; Maryland; Talbot County; Easton |
| NMNH | 598291 | 285.047 | 21.6 | USA; Maryland; Talbot County; St. Michaels |
| NMNH | 598292 | 264.129 | 21.4 | USA; Maryland; Talbot County; St. Michaels |
| NMNH | 598293 | 311.564 | 22.547 | USA; Maryland; Talbot County; St. Michaels |
| NMNH | 598294 | 294.31 | 22.71 | USA; Maryland; Talbot County; St. Michaels |
| NMNH | 598295 | 320.652 | 22.09 | USA; Maryland; Talbot County; St. Michaels |
| NMNH | 598296 | 293.292 | 21.443 | USA; Maryland; Talbot County; St. Michaels |
| NMNH | 598297 | 258.46 | 21.15 | USA; Maryland; Talbot County; St. Michaels |
| NMNH | 598298 | 285.116 | 21.38 | USA; Maryland; Talbot County; St. Michaels |
| NMNH | 598299 | 297.872 | 23.343 | USA; Maryland; Talbot County; St. Michaels |
| NMNH | 598300 | 294.258 | 22.417 | USA; Maryland; Talbot County; St. Michaels |
| NMNH | 598301 | 290.555 | 21.567 | USA; Maryland; Talbot County; St. Michaels |
| NMNH | 598302 | 275.992 | 20.457 | USA; Maryland; Talbot County; St. Michaels |
| NMNH | 598303 | 310.493 | 22.043 | USA; Maryland; Talbot County; St. Michaels |
| NMNH | 598304 | 275.203 | 22.357 | USA; Maryland; Talbot County; St. Michaels |
| NMNH | 598305 | 271.885 | 23.177 | USA; Maryland; Talbot County; St. Michaels |
| NMNH | 598306 | 293.25 | 21.273 | USA; Maryland; Talbot County; Royal Oak |
| NMNH | 598307 | 318.379 | 22.193 | USA; Maryland; Talbot County; Royal Oak |
| NMNH | 598308 | 275.955 | 20.663 | USA; Maryland; Talbot County; Royal Oak |
| NMNH | 598309 | 262.813 | 21.55 | USA; Maryland; Talbot County; Newcomb |
| NMNH | 598310 | 304.65 | 21.41 | USA; Maryland; Talbot County; Royal Oak |
| NMNH | 598311 | 298.004 | 22.247 | USA; Maryland; Talbot County; Royal Oak |
| NMNH | 598312 | 281.41 | 21.723 | USA; Maryland; Talbot County; Royal Oak |
| NMNH | 598314 | 270.069 | 20.897 | USA; Maryland; Queen Annes County; Grasonville |
| NMNH | 598315 | 298.27 | 21.743 | USA; Maryland; Talbot County; McDaniel |
| NMNH | 598321 | 285.136 | 21.503 | USA; Maryland; Talbot County; Wye Mills |
| NMNH | 598322 | 313.076 | 23.433 | USA; Maryland; Talbot County; Wittman |
| NMNH | 598323 | 321.783 | 21.48 | USA; Maryland; Talbot County; Trappe |
| NMNH | 598324 | 251.458 | 21.303 | USA; Maryland; Talbot County; Oxford |
| NMNH | 598325 | 272.556 | 20.55 | USA; Maryland; Talbot County; St. Michaels |
| NMNH | 598326 | 240.102 | 20.763 | USA; Maryland; Talbot County; St. Michaels |
| NMNH | 598327 | 276.763 | 21.253 | USA; Maryland; Talbot County; St. Michaels |
| NMNH | 598328 | 250.256 | 22.55 | USA; Maryland; Talbot County; St. Michaels |
| NMNH | 598329 | 282.027 | 20.703 | USA; Maryland; Talbot County; St. Michaels |
| NMNH | 598330 | 289.956 | 21.98 | USA; Maryland; Talbot County; St. Michaels |
| NMNH | 598331 | 315.421 | 22.227 | USA; Maryland; Talbot County; Easton |
| NMNH | 598333 | 308.094 | 21.847 | USA; Maryland; Talbot County; Easton |
| NMNH | 598335 | 296.138 | 21.523 | USA; Maryland; Talbot County; Easton |
| NMNH | 598338 | 262.796 | 20.997 | USA; Maryland; Kent County; Rock Hall |
| NMNH | 598341 | 288.323 | 20.433 | USA; Maryland; Talbot County; Easton |
| NMNH | 598342 | 311.66 | 21.183 | USA; Maryland; Talbot County; Easton |
| NMNH | 598343 | 261.936 | 20.4 | USA; Maryland; Talbot County; Easton |
| NMNH | 598344 | 292.968 | 21.287 | USA; Maryland; Talbot County; Easton |
| NMNH | 598345 | 281.451 | 21.563 | USA; Maryland; Talbot County; Easton |
| NMNH | 598346 | 287.263 | 22.33 | USA; Maryland; Talbot County; Easton |
| NMNH | 598347 | 288.698 | 22.787 | USA; Maryland; Talbot County; St. Michaels |
| NMNH | 598348 | 266.788 | 18.69 | USA; Maryland; Talbot County; St. Michaels |
| NMNH | 598349 | 259.56 | 21.287 | USA; Maryland; Talbot County; St. Michaels |
| NMNH | 598352 | 302.538 | 23.183 | USA; Maryland; Talbot County; Oxford |
| NMNH | 598353 | 279.387 | 23.293 | USA; Maryland; Talbot County; Trappe |
| NMNH | 599840 | 300.237 | 21.097 | USA; Maryland; Talbot County; Royal Oak |
| NMNH | 599841 | 279.994 | 20.3 | USA; Maryland; Talbot County; Bozman |
| NMNH | 601533 | 324.901 | 20.54 | USA; Virginia; Ceres County; Bland |
| NMNH | 602334 | 316.634 | 22.663 | USA; Maryland; Queen Annes County; Queenstown |
| NMNH | 626495 | 267.281 | 21.06 | USA; Florida; Okaloosa County; Eglin |
| UWZM | A20784 | 307.928 | 25.4 | USA; Wisconsin; Jefferson County; Jefferson |
| UWZM | A20785 | 290.792 | 21.547 | USA; Wisconsin; Waukesha County; Madison |
| UWZM | A20787 | 282.408 | 22.727 | USA; Wisconsin; Dane County; Marschall |
| UWZM | A20788 | 277.514 | 23.253 | USA; Wisconsin; Rock County; Milton |
| UWZM | A20791 | 296.122 | 23.577 | USA; Wisconsin; Dane County; Madison |
| UWZM | A20792 | 283.463 | 23.273 | USA; Wisconsin; Sauk County; Baraboo |
| UWZM | A23085 | 330.412 | 23.31 | USA; Wisconsin; Dane County; Madison |
| UWZM | A23502 | 327.653 | 24.047 | USA; Wisconsin; Dane County; Westport Township |
| UWZM | A23989 | 292.932 | 23.87 | USA; Wisconsin; Dane County; Madison |

Table S2. Results of the model of bill surface area across the United States for female and male northern cardinals. Predictor variables include tarsus length, year, average minimum temperature (mintemp), relative humidity (hum) and housing density (hden), against which bill size was regressed. We present the parameter estimate, standard error of that estimate, and the p-value.

|  | *All Birds** |  |  |
| --- | --- | --- | --- |
| Variable | Estimate | Standard Error | P-Value |
| Tarsus | 2.052 | 1.658 | 0.217 |
| Year | 0.687 | 1.677 | 0.682 |
| Mintemp | 2.485 | 1.777 | 0.163 |
| Hum | -8.721 | 1.914 | <0.001 |
| Hden | -3.596 | 1.438 | 0.013 |
| Mintemp x Hum | 4.078 | 1.923 | 0.035 |
| Mintemp x Hden | -2.044 | 1.183 | 0.085 |
|  | *Females** |  |  |
| Variable | Estimate | Standard Error | P-Value |
| Tarsus | -0.509 | 3.043 | 0.868 |
| Year | 0.047 | 0.101 | 0.643 |
| Mintemp | 5.064 | 3.299 | 0.128 |
| Hum | -1.367 | 4.727 | 0.773 |
| Hden | -2.723 | 2.345 | 0.248 |
| Mintemp x Hum | 0.390 | 3.581 | 0.914 |
| Mintemp x Hden | 0.583 | 3.609 | 0.872 |
|  | *Male** |  |  |
| Variable | Estimate | Standard Error | P-Value |
| Tarsus | 2.726 | 1.882 | 0.149 |
| Year | -0.006 | 0.071 | 0.931 |
| Mintemp | 4.151 | 2.097 | 0.049 |
| Hum | -10.132 | 2.037 | <0.001 |
| Hden | -3.008 | 1.733 | 0.084 |
| Mintemp x Hum | 1.069 | 2.504 | 0.670 |
| Mintemp x Hden | -1.806 | 1.281 | 0.160 |

Table S3. Model results for female and male northern cardinals over time. We present results for each subregion and included predictors of average minimum temperature (mintemp), housing density (hden) and year. Tarsus is included in all models as a predictor. Female bill size in Ithaca, New York and Washington D.C. increased with increasing mintemp. No temporal relationships are evident for male bill size. Model descriptors include the parameter estimate, standard error of that estimate, and the p-value.

|  |  | *Washington D.C.* |  | |  |
| --- | --- | --- | --- | --- | --- |
| Location | Parameter* | Estimate | Standard Error | | P-Value |
| *All Birds* |  |  |  |  | |
|  | Mintemp | 7.754 | 8.939 | | 0.391 |
|  | Tarsus | 5.489 | 2.778 | | 0.055 |
|  | Year | 0.108 | 0.664 | | 0.872 |
|  | Tarsus | 5.652 | 2.945 | | 0.062 |
|  | Hden | 1.964 | 2.778 | | 0.484 |
|  | Tarsus | 5.600 | 2.778 | | 0.051 |
| *Females* |  |  |  | |  |
|  | Mintemp | 14.683 | 14.510 | | 0.327 |
|  | Tarsus | 4.316 | 4.980 | | 0.399 |
|  | Year | 1.918 | 1.182 | | 0.124 |
|  | Tarsus | 3.159 | 4.775 | | 0.518 |
|  | Hden | 11.018 | 3.873 | | 0.012 |
|  | Tarsus | 5.448 | 3.835 | | 0.175 |
| *Males* |  |  |  | |  |
|  | Mintemp | 1.179 | 13.118 | | 0.929 |
|  | Tarsus | 5.621 | 4.301 | | 0.205 |
|  | Year | -0.837 | 0.780 | | 0.295 |
|  | Tarsus | 6.872 | 4.347 | | 0.129 |
|  | Hden | -5.214 | 3.532 | | 0.155 |
|  | Tarsus | 7.005 | 4.201 | | 0.110 |
|  |  | *Chicago* |  | |  |
| Location | Parameter* | Estimate | Standard Error | | P-Value |
| *All Birds* |  |  |  | |  |
|  | Mintemp | -12.387 | 7.636 | | 0.115 |
|  | Tarsus | -2.500 | 3.377 | | 0.465 |
|  | Year | -0.248 | 0.162 | | 0.137 |
|  | Tarsus | -1.713 | 3.413 | | 0.619 |
|  | Hden | -8.247 | 4.047 | | 0.057 |
|  | Tarsus | 1.081 | 3.627 | | 0.769 |
| *Females* |  |  |  | |  |
|  | Mintemp | -7.281 | 8.788 | | 0.429 |
|  | Tarsus | -3.684 | 5.603 | | 0.527 |
|  | Year | -0.207 | 0.199 | | 0.325 |
|  | Tarsus | -2.865 | 5.483 | | 0.614 |
|  | Hden | -12.333 | 5.703 | | 0.083 |
|  | Tarsus | 0.765 | 4.776 | | 0.879 |
| *Males* |  |  |  | |  |
|  | Mintemp | -19.765 | 13.351 | | 0.156 |
|  | Tarsus | -2.399 | 4.423 | | 0.594 |
|  | Year | -0.429 | 0.266 | | 0.124 |
|  | Tarsus | -1.853 | 4.398 | | 0.679 |
|  | Hden | -7.227 | 5.544 | | 0.222 |
|  | Tarsus | 0.991 | 5.091 | | 0.850 |
|  |  | *Ithaca* |  | |  |
| Location | Parameter* | Estimate | Standard Error | | P-Value |
| *All Birds* |  |  |  | |  |
|  | Mintemp | -0.111 | 6.759 | | 0.987 |
|  | Tarsus | 2.276 | 3.271 | | 0.495 |
|  | Year | -0.005 | 0.191 | | 0.980 |
|  | Tarsus | 2.262 | 3.329 | | 0.505 |
|  | Hden | -0.333 | 3.364 | | 0.922 |
|  | Tarsus | 2.199 | 3.364 | | 0.521 |
| *Females* |  |  |  | |  |
|  | Mintemp | 5.418 | 16.919 | | 0.765 |
|  | Tarsus | -6.475 | 7.320 | | 0.426 |
|  | Year | 0.047 | 0.724 | | 0.951 |
|  | Tarsus | -7.774 | 6.184 | | 0.277 |
|  | Hden | -2.820 | 4.681 | | 0.579 |
|  | Tarsus | -8.877 | 5.883 | | 0.206 |
| *Males* |  |  |  | |  |
|  | Mintemp | -3.971 | 6.989 | | 0.580 |
|  | Tarsus | 4.646 | 3.441 | | 0.202 |
|  | Year | 0.121 | 0.195 | | 0.546 |
|  | Tarsus | 4.974 | 3.501 | | 0.181 |
|  | Hden | 1.999 | 3.846 | | 0.613 |
|  | Tarsus | 4.974 | 3.549 | | 0.186 |

**Note: Minimum temperature, housing density and year were included in separate linear models. Tarsus was included in each model as a proxy for body size.*

Table S4. Linear-mixed model conducted for female and male Northern Cardinals. Predictor variables include year, average maximum temperature (maxtemp), relative humidity (hum), and housing density (hden) which were regressed against bill size. Model descriptors include the parameter estimate, standard error of that estimate, and the p-value.

|  | ***All Birds*** |  |  |
| --- | --- | --- | --- |
| **Variable** | **Estimate** | **Standard Error** | **P-Value** |
| Year | -0.966 | 1.586 | 0.543 |
| Maxtemp | 1.164 | 1.700 | 0.494 |
| Hum | -9.901 | 2.086 | 0.000 |
| Hden | -4.407 | 1.583 | 0.006 |
| Maxtemp x Hum | 4.976 | 2.003 | 0.014 |
| Maxtemp x Hden | -2.687 | 1.288 | 0.038 |
|  | ***Females*** |  |  |
| **Variable** | **Estimate** | **Standard Error** | **P-Value** |
| Year | -0.046 | 0.098 | 0.640 |
| Maxtemp | 0.819 | 3.104 | 0.792 |
| Hum | -7.183 | 5.313 | 0.179 |
| Hden | -4.008 | 2.550 | 0.119 |
| Maxtemp x Hum | 4.012 | 4.070 | 0.326 |
| Maxtemp x Hden | -2.035 | 3.492 | 0.561 |
|  | ***Males*** |  |  |
| **Variable** | **Estimate** | **Standard Error** | **P-Value** |
| Year | -0.064 | 0.065 | 0.329 |
| Maxtemp | 2.767 | 1.980 | 0.164 |
| Hum | -10.497 | 2.189 | 0.000 |
| Hden | -3.874 | 1.915 | 0.044 |
| Maxtemp x Hum | 2.042 | 2.450 | 0.406 |
| Maxtemp x Hden | -2.460 | 1.425 | 0.086 |

**Appendix Figure Legends:**

Figure A1: Box and whisker plot depicting bill size distributions of male and female cardinals. The notches represent confidence intervals, the lines represent 1.5 times the inter-quartile range, and the dots represent cardinals lying outside of this range. Female cardinals have significantly smaller bills than male cardinals, a pattern which was true across the full Northern Cardinal range. BSA stands for bill surface area.

**Figure A1.**


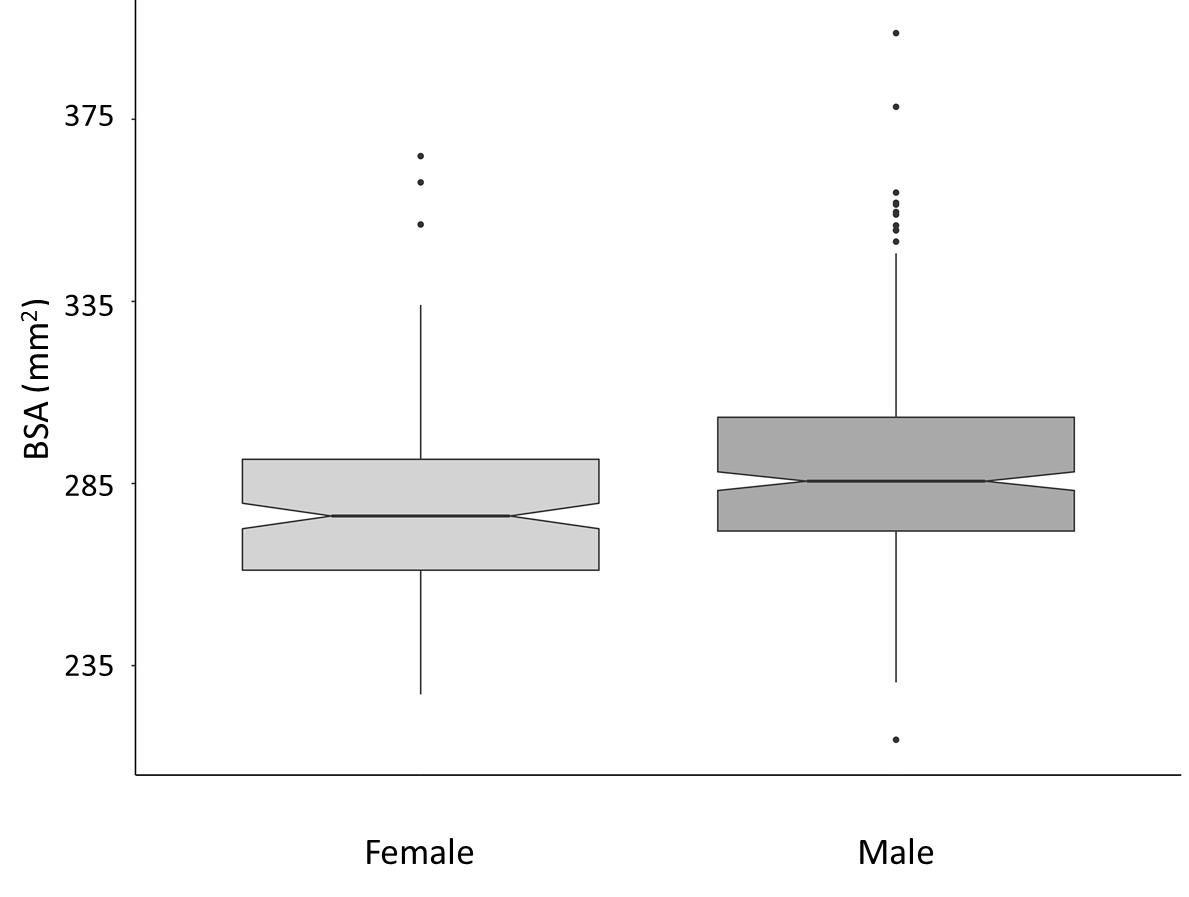

Supplement: Supplementary file 1 [file ECE3-8-4841-s001.docx]
